# CAM-DR: Mechanisms, Roles and Clinical Application in Tumors

**DOI:** 10.3389/fcell.2021.698047

**Published:** 2021-07-06

**Authors:** Yuejiao Huang, Yuchan Wang, Jie Tang, Shiyi Qin, Xianjuan Shen, Song He, Shaoqing Ju

**Affiliations:** ^1^Medical School, Nantong University, Nantong, China; ^2^Department of Medical Oncology, Affiliated Tumor Hospital of Nantong University, Nantong, China; ^3^Department of Pathogenic Biology, School of Medicine, Nantong University, Nantong, China; ^4^Department of Laboratory Medicine, Affiliated Hospital of Nantong University, Nantong, China; ^5^Department of Pathology, Affiliated Tumor Hospital of Nantong University, Nantong, China

**Keywords:** CAM-DR, tumor microenvironment, hematologic malignancies, signaling pathways, inhibitors

## Abstract

Despite the continuous improvement of various therapeutic techniques, the overall prognosis of tumors has been significantly improved, but malignant tumors in the middle and advanced stages still cannot be completely cured. It is now evident that cell adhesion-mediated resistance (CAM-DR) limits the success of cancer therapies and is a great obstacle to overcome in the clinic. The interactions between tumor cells and extracellular matrix (ECM) molecules or adjacent cells may play a significant role in initiating the intracellular signaling pathways that are associated with cell proliferation, survival upon binding to their ligands. Recent studies illustrate that these adhesion-related factors may contribute to the survival of cancer cells after chemotherapeutic therapy, advantageous to resistant cells to proliferate and develop multiple mechanisms of drug resistance. In this review, we focus on the molecular basis of these interactions and the main signal transduction pathways that are involved in the enhancement of the cancer cells’ survival. Furthermore, therapies targeting interactions between cancer cells and their environment to enhance drug response or prevent the emergence of drug resistance will also be discussed.

## Introduction

Chemotherapy remains the major treatment of disseminated cancer including hematologic malignancies and metastatic solid tumors. Intrinsic and acquired resistance disseminated continues to be a considerable obstacle on our way to improve patient cures. Resistance mechanisms originate from pathological changes of intrinsic factors such as genetics, epigenetics, transcriptional regulation, and protein activation as well as extrinsic factors including the immune system, hypoxia, metabolism, and extracellular matrix (ECM). It was proposed that the tumor microenvironment, the non-cancerous cells, and ECM that are in direct contact with the cancer cell, may influence how cancer cells respond to chemotherapy. This influence mainly involves two aspects: a soluble factor and a contact factor. The first aspect involves soluble mediators, such as interleukins, that are secreted by non-tumor, stromal cells. It is capable of providing further signals for tumor cell growth and survival. The second aspect of tumor cell-environment interaction requires direct cell contact and has been given the term cell-adhesion-mediated drug resistance (CAM-DR). Recently, attention has been focused mainly on the effect of CAM-DR and cell adhesion to ECM proteins has been well clarified as a key and fundamental determinant of cancer therapy resistance. Adhesive interactions between cells or cells and ECM can influence cell behavior and tumor cell adhesion may influence cell survival and prevent drug-induced apoptosis. In this review, we will describe the current knowledge about cell adhesion resistance and put these findings into a clinical context and discuss the resulting consequences for the design of novel therapeutic strategies.

## Microenvironment and CAM-DR

**FIGURE 1:**
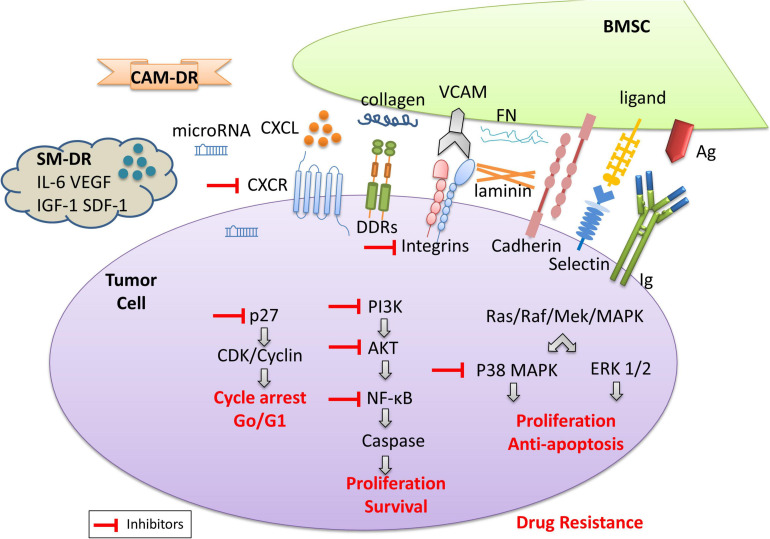
The model diagram of the tumor microenvironment. The cross-talk between tumor cells and stromal cells is regulated by different mechanisms: (i) cell-to-cell adhesion between tumor cells and ECM components (i.e., collagens, fibronectin, and laminin)/stromal cells; and (ii) soluble factors, i.e., cytokines, chemokines, growth factors, exosomes, and miRNAs released by the stromal cells and MM cells, with autocrine and paracrine effects. The adhesion of tumor cells and stromal cells is associated with cell surface antigen and antibodies, the expression of the integrin family, discoidin-domain receptors (DDR), cadherin, selectin, etc. As shown here, collagen and other proteins are involved in CAM-DR, while soluble cytokines are usually involved in SM-DR. They contribute to drug-resistance upon engagement with their ligands in the tumor microenvironment. The activation of the signaling pathways involved leads to tumor cell proliferation, anti-apoptosis, and survival, resulting in drug resistance. Inhibitors of related signaling pathways are beneficial to the reversal of drug resistance.

The tumor microenvironment includes a non-cellular compartment formed by ECM proteins (i.e., laminin, fibronectin, and collagen) and soluble factors (i.e., cytokines, growth factors, chemokines, exosomes, and miRNAs), and a rich cellular compartment constituted by tumor cells and stromal cells (i.e., fibroblasts, osteoclasts, endothelial cells, and mesenchymal stromal cells) (Manier et al., 2012) (Figure 1). The tumor microenvironment provides support for the growth, proliferation, and invasion of tumor cells through intercellular connections and molecular interactions. Moreover, the tumor microenvironment plays an important role in drug resistance (Burger et al., 2009). Studies have found that hematologic malignant tumor cells die rapidly *in vitro*, which confirms the importance of the tumor microenvironment for tumor cell survival (Li and Dalton, 2006). A growing body of experimental data shows that hematological tumor cells can survive significantly longer when co-cultured with stromal cells *in vitro*. Within or surrounding the malignant tumor cells, stromal cells, lymphocytes, and endothelial cells are present which interact with each other and/or with the tumor cells. Besides, soluble factors in the tumor microenvironment provide signals for cell growth and survival. The cross-talk between tumor cells and stromal cells is regulated by different mechanisms: (i) cell-to-cell adhesion between tumor cells and ECM components/stromal cells; and (ii) soluble factors, released by the stromal cells and MM cells, with autocrine and paracrine effects (Di Marzo et al., 2016). Thus, microenvironment mediated-drug resistance (EM-DR) in tumor cells can generally be divided into the intrinsic soluble factor mediated-drug resistance (SM-DR) and cell adhesion mediated-drug resistance (CAM-DR).

It has been reported that many soluble factors in the tumor microenvironment are related to the growth and survival of cells, such as vascular growth factor (VEGF) (Dias et al., 2002), interleukin-3, 6 (IL-3; IL-6) (Lee et al., 2004; Karakasheva et al., 2018), nitric oxide (NO) (Vedenko et al., 2020), granulocyte colony-stimulating factor (G-CSF) (Karagiannidis et al., 2021), B cell-activating factor of the TNF family (BAFF) (Lwin et al., 2009), etc. These soluble factors can not only stimulate the proliferation and survival of tumor cells but also lead to drug resistance. However, inhibition of these known soluble factor-mediated cell survival pathways does not overcome the phenomenon of drug resistance. Drug resistance mechanisms are multifaceted, such as gene mutations, cell cycle arrest, or CAM-DR through direct cell-to-cell contact and adhesion (Oshiro et al., 2001).

Compared with SM-DR in the tumor microenvironment, CAM-DR plays a greater role in the drug resistance of tumor cells. On the one hand, cell adhesion activates key signaling pathways leading to the expression of soluble factors and their receptors. For example, the interaction between hematologic tumor cells and stromal cells stimulates bone marrow stromal cells (BMSCs) to secrete IL-6 and granulocyte-macrophage colony-stimulating factor (GM-CSF), while MM tumor cells secrete IL-6 and VEGF, etc. (Dalton, 2003). On the other hand, cell adhesion can lead to changes that cannot be caused by soluble factors. For example, reactivation of the expression of key molecules involved in drug resistance, such as the anti-apoptotic molecule c-FLIP_*L*_, leads to Fas-mediated apoptosis (Shain et al., 2002). Another example is topoisomerase II-β, a key enzyme involved in the repair of DNA double-strand breaks, which can lead to drug resistance of tumor cells (Hazlehurst et al., 2001; Said et al., 2012). It also includes the increased expression of B cell lymphoma-2 (Bcl-2) and the activation of downstream signaling pathways (Yan et al., 2015), and so on, which represents a new mechanism of drug resistance. Cell adhesion is mediated by several families of adhesion molecules, including the immunoglobulin superfamily, integrins, cadherins, and selectins (Kim et al., 2020). The tumor microenvironment is abundant in binding partners for cell adhesion molecules as each component discussed above expresses diverse ligands and secret ECM (Malara et al., 2014; Galan-Diez et al., 2018; Zhao et al., 2019) (Figure 1). Fibronectin (FN), collagen, and laminin will interact with cell surface molecules (Zhang et al., 2019). Integrins are the most widely studied molecule at present (Elliott and Sethi, 2002). Integrin is a heterodimer receptor composed of two subunits α and β. Up to now, 18 α subunits and 8 β subunits have been identified (Miranti and Brugge, 2002). The expression of typical integrins is mainly α4β1 (integrin very late antigen-4, VLA-4) and α5β1 (VLA-5) in hematologic malignancies, especially VLA-4 is highly expressed in multiple myeloma (MM) cells and most normal B lymphocytes (Mraz et al., 2011). In myeloma cells, overexpression of VLA-4 can subsequently lead to increased drug resistance, whereas the reversal a drug resistance is usually associated with decreased VLA-4 expression (Fontana et al., 2021; Sevilla-Movilla et al., 2020). Most integrins bind to ECM proteins (Hynes, 1992), among which the most important of which is fibronectin, which is the ligand of at least 10 integrin molecules. Cadherins can be classified into several different types including Type I and Type II classical cadherins (Ivanov et al., 2001). Stromal cells upregulate cadherin expression in leukemia cell lines and increase resistance to imatinib by stabilizing β-catenin (Chen et al., 2014). When interrupted the N-cadherin-mediated adhesion of chronic myeloid leukemia (CML) cells to BMSCs, the tumor cells gained sensitivity toward imatinib treatment (Zhang et al., 2013). Selectins (CD62) are single-chain transmembrane glycoproteins that mediate calcium-dependent carbohydrate-binding (Borsig, 2018). Hematological tumor cells interact with E-selectin through various ligands such as CD43, CD44, and PSGL-1 (Bistrian et al., 2009). Although relatively specific ligands are preferentially bound in different cells, this does not mean that other ligands are less crucial in the CAM-DR of the disease. Interestingly, nilotinib treatment upregulated the expression of E-selectin, which may result in the increased adherence of leukemia cells to E-selectin and the evasion of the cytotoxicity induced by chemotherapy (Hadzijusufovic et al., 2017). Discoidin domain receptors (DDR), including DDR1 and DDR2, are special types of the transmembrane receptor tyrosine kinase superfamily. DDR are activated by binding to the collagen and can activate signal transduction pathways. At the same time, DDR can regulate cell-collagen interactions which involved in multiple processes such as cell proliferation, migration, and apoptosis (Gao et al., 2021). Moreover, the IIGFs-DDR1 crosstalk is considered the major mediator of therapy resistance of cancer cells (Buck et al., 2010; Vella et al., 2019). The model diagram of tumor microenvironment is shown in Figure 1.

**FIGURE 2:**
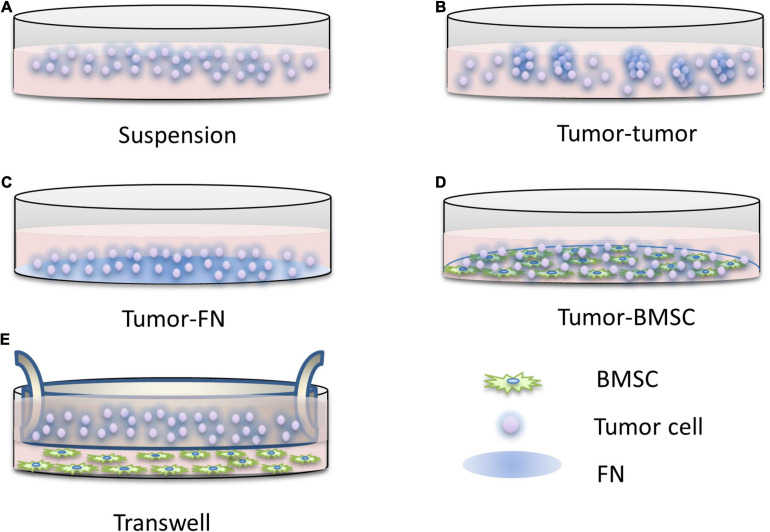
The research pattern diagram of CAM-DR. Construction of CAM-DR model *in vitro* through the construction of FN adhesion model **(C)** and cell–cell direct adhesion model **(D)**, transwell model **(E)** can be used to study the relationship of directly acting soluble factors between cells, organ-like culture model **(B)** is relatively less used, and simple suspension culture model **(A)** is used as control.

On this basis, the simplified model of the tumor microenvironment is to construct the FN model, that is, the interaction between tumor cells and FN leads to CAM-DR, which is convenient for the study of drug resistance caused by the adhesion of tumor cells to the ECM. At present, the models of CAM-DR have been widely used to simulate the interactions between tumor cells and tumor cells, tumor cells and ECM, and tumor cells and stromal cells. In addition to the FN model, there are also stromal models, which can be used to study CAM-DR and SM-DR. First proposed by the Dalton Laboratory, FN model allows the researchers to investigate the specific signaling pathways of CAM-DR which involved in cell adhesion associated with FN or β1 integrin activation (Hazlehurst and Dalton, 2001), elucidating the role of two major adhesion molecules, VLA-4 and VLA-5, in hematological malignancies. One of the most typical findings of this model is that the activation of NF-κB signaling by FN is associated with the occurrence of CAM-DR (Landowski et al., 2003). Using the FN model, it was found for the first time that the down-regulation of the apoptotic protein Bim in myeloma was closely related to CAM-DR. This finding also proved that the integrin-mediated signaling pathway in myeloma is similar to the role of EGFR in the EGFR signaling pathway and can regulate the expression of Bim protein (Hazlehurst et al., 2003). FN adhesion model can also cause cell cycle arrest in the G1 phase, increase the expression of cell cycle inhibitor p27 (Lwin et al., 2007a), and inhibit the activity of CDK2 (Jiang et al., 2000b), a key molecule of cell division. Clinically related studies have found that G1 phase arrest of the cell cycle can significantly reduce the efficacy of drugs (Jiang et al., 2000a). These findings also explain the rapid proliferation of tumor cells in the microenvironment and their insensitivity to cytotoxic drug killing mechanisms. However, the FN model ignores the fact that stromal cells play a more complex role in the tumor microenvironment than fibronectin. The matrix model constructed by the cell compartment culture system can enable researchers to eliminate the interference of SM-DR in the study of CAM-DR, so it creates the condition for the study of CAM-DR *in vitro*. Similar results were found using the same model as the FN model (Said et al., 2012). Mudry et al. (2000) found that direct interaction with stromal cells, rather than the action of soluble factors, protects leukemic cells from drug-induced apoptosis. Blockage of the vascular cell adhesion molecule-1 (VCAM-1) signaling pathway by anti-VCAM reagents abolished this protective effect, while the FN model was unable to identify whether VLA-4-mediated interactions with VCAM. The drug resistance caused by this interaction is associated with down-regulation of the pro-apoptotic molecule caspase-3 (Fortney et al., 2001). In other studies, co-culture of myeloma cells with matrix in conditioned media with soluble factors, rather than just conditioned stromal cells, protected myeloma cells from mitoxantrone drug-mediated apoptosis (Nefedova et al., 2003). However, the cell compartment culture of stromal cells and myeloma cells also produced the phenomenon of drug apoptosis protection, which further supports the interaction between tumor cells and stromal cells as an important part of drug resistance. Although the mechanism of this has not been thoroughly studied, it can be concluded that the adhesion of tumor cells to stromal cells is inseparable from cell cycle arrest and so on to avoid drug-induced apoptosis. The model diagram of CAM-DR research is shown in Figure 2.

In general, the interaction between the tumor microenvironment and tumor cells provides survival signals for tumor cells, regardless through direct contact or the production of soluble factors in the microenvironment. They can up-regulate anti-apoptotic molecules, down-regulate pro-apoptotic molecules, promote cell proliferation, reduce DNA damage, and increase DNA repair ability through different signaling pathways (Dehghanifard et al., 2018; Mensah et al., 2018). Therefore, the tumor microenvironment plays an integral role in tumor cell survival and drug response. The intervention of the factors that promote the growth, survival, angiogenesis, and drug resistance of tumor cells in the microenvironment will be a new therapeutic approach in the future (Naci et al., 2015). Some of these drugs have been approved for clinical use (such as Idelalisib and Dactolisib, etc.), while the others are still in clinical trials (such as Buparlisib, Dactolisib, etc.). Related inhibitors based on pre-clinical research provide new insights into the treatment of cancer and this might bring new bright prospects (Sanchez et al., 2019). The sulfonamide derivative E7820, which inhibits α2 integrin subunit gene expression is used in combination with chemotherapy in phase II clinical trials in patients with various advanced or refractory malignancies (Keizer et al., 2011; Mita et al., 2011). A humanized blocking mAb against 2 integrin chains (GBR-500, Glenmark-Sanofi) has been developed and was well tolerated in phase I clinical trials. GBR-500 can block the local growth of human prostate cancer cell lines *in vivo* and increases the sensitivity of hepatocarcinoma cell lines to cytotoxic drugs (Naci et al., 2015). Targeted drugs for tumor cells and tumor microenvironment can eliminate or reduce small residual lesions of tumors and reduce acquired drug resistance.

## Roles of CAM-DR in Tumors

### Hematologic Malignancies

#### Multiple Myeloma (MM)

Multiple myeloma is the second most common hematological malignancy, accounting for about 10% of all hematological malignancies. It is characterized by abnormal proliferation of plasma cells, secretion of a monoclonal immunoglobulin or its fragments (M protein), resulting in the related organ or tissue damage. The occurrence and development of myeloma are closely related to some special changes of tumor cells, such as genomic/chromosomal instability, gene mutations, chromosome translocations, and the interaction of ECM, that is, the continuous dynamic interaction between myeloma cells and bone marrow microenvironment (Di Marzo et al., 2016). The treatment of myeloma is mainly targeted at tumor cells by chemotherapy and targeted therapy, stem cell transplantation, and symptomatic treatment. However, the 5-year survival rate of MM is about 40 months from 1990 to 2000. With the continuous development and clinical application of new drugs such as bortezomib, carfilzomib, lenalidomide, pomalidomide, and so on, the 5-year survival rate of MM has increased significantly since the beginning of the 21st century, reaching about 60 months (Furukawa and Kikuchi, 2016). Although the therapeutic drugs for MM are constantly developing, drug resistance is an important cause of death, resulting in poor prognosis, recurrence, and death. Existing studies have shown that the interaction of myeloma cells and tumor microenvironment plays an important role in the treatment of drug resistance, especially CAM-DR (Di Marzo et al., 2016; Ullah, 2019). The role of the bone marrow microenvironment is to produce a specific phenotype through the regulation of cytokines and direct contact with MM cells, resulting in insensitivity to treatment.

The molecules involved in CAM-DR in MM are commonly found in some chemokine receptors and integrins, and some cell cycle regulatory factors and apoptosis-related proteins can also be seen. The C-X-C motif chemokine receptors (CXCR) involved in CAM-DR include CXCR4, CXCR7, CXCR12 (Waldschmidt et al., 2017; Wang et al., 2019). They are classical adhesion molecules and homing factors, which are usually highly expressed on the surface of MM cells or BMSCs and can regulate cell-to-cell adhesion independently or mutually. Many upstream molecules usually regulate CAM-DR by regulating chemokine. However, MM can develop from asymptomatic to symptomatic stages, including monoclonal gammopathy of unknown significance (MGUS), smoldering multiple myeloma (SMM), MM, or plasma cell leukemia (Willenbacher et al., 2018). At the same time, CXCR is usually involved not only in CAM-DR but also in the regulation of the biological behavior of other cells, such as the maturation and development of MM cells mentioned above (Ullah, 2019). Studies have shown that in the tumor microenvironment, the adhesion of tumor cells to stromal cells can promote stromal cells to secrete high levels of IL-6, tumor necrosis factor-α, and osteopontin as ligands of integrins to bind and activate integrins (Damiano et al., 1999). Previous studies indicated that integrin β1 was highly expressed on the cell membrane in MM, and the adhesion of MM cells to ECM mediated by integrin β1 could significantly inhibit the apoptosis signal of caspase cells induced by chemotherapeutic drugs (Rainero and Norman, 2015; Huang et al., 2019). Hazlehurst et al. (2000) also found that integrin β1 mediated cell adhesion to ECM cells significantly increased the protein level of cell cycle inhibitory protein p27^*Kip*1^, which blocked cells in the G0/G1 phase, thus escaping the killing effect of toxic drugs. The progression of tumors and the emergence of drug resistance are closely related to the NF-κB signaling pathway (Keats et al., 2007). The NF-κB signaling pathway can be activated by cytokines in the tumor cell microenvironment, or by binding to FN and secreting related factors (Landowski et al., 2003). By blocking the typical/atypical NF-κB pathway and hindering the degradation of Iκβ protein, bortezomib has become a standard drug in MM therapy (Murray and Norbury, 2000; Gilmore, 2007).

The adhesion of the bone marrow microenvironment in MM not only affects the apoptosis pathway and cell cycle but also leads to some other mechanisms of CAM-DR, including epigenetic mechanisms (Furukawa and Kikuchi, 2016). Previous studies have found that class I histone deacetylases (HDACs) can detect the sensitivity of proteasome inhibitors, and histone methyltransferase EZH2 can regulate the transcriptional activity of anti-apoptotic proteins activated in CAM-DR (Kikuchi et al., 2015). Besides, another histone methyltransferase MMSET can promote the repair of DNA damage and lead to drug resistance (Brito et al., 2009). With more and more in-depth research, more molecular markers have been discovered and studied in the CAM-DR of MM, including microRNAs (miRNAs/miRs) (Wang et al., 2011; Wu et al., 2019), exosomes, and cancer-associated fibroblasts (CAFs) (Di Marzo et al., 2016). The disruption of fibronectin-heparan sulfate interactions blocks exosome binding to MM cells or BM stromal cells, highlighting a specific cross-talk fostered by exosomes in the bone marrow microenvironment (Thompson et al., 2013). CAFs can also serve the function of modifying the bone marrow stroma, influencing chemotaxis, adhesion, proliferation, and apoptosis caused by CAM-DR and the expression of adhesion molecules (Frassanito et al., 2014). This article summarizes the CAM-DR of MM (see Table 1).

**TABLE 1:** CAM-DR in MM.

Molecules	Expression level	Biological functions	Signaling pathways	References
ANXA7/CDC5L	Up	Promote the cell cycle, proliferation, and CAM-DR		Liu et al., 2020
BTK/CXCR4	Up	Relate to MM progression and drug resistance		Wang et al., 2019
p-EZH2	Up	p-EZH2(S21) induce hypermethylation of H3K27, lead to the sustained expression of antiapoptotic genes	IGF-1R/PI3K/AKT	Kikuchi et al., 2015
CXCL12/CXCR7	Up	Enhance CAM-DR	ERK/MAPK	Waldschmidt et al., 2017
Integrin α 6β 1	Up	An autocrine mechanism in CAM-DR	Wnt3/RhoA/ROCK	Kobune et al., 2007
Integrin β 7	Up	Enhance CAM-DR, migration, and BM homing	NF-κB	Neri et al., 2011
Integrin α 4β 1	Up	CAM-DR and inhibit cell apoptosis	NF-κB	Fontana et al., 2021; Sevilla-Movilla et al., 2020
Numbl/Integrin β 1	Up	Regulate cell-cycle progression	PI3K/AKT	Huang et al., 2019
p27	Up	Regulate cell-cycle progression		Hazlehurst et al., 2000; Zhan et al., 2007
PDCD4	Down	Confer drug resistance via enhancing AKT phosphorylation at Ser473		Wu et al., 2019
CHD1L AGS3	Up	Anti-apoptotic	Caspase-9/3	Shao et al., 2014; Xu et al., 2016
EphA4	Up	Promote proliferation via the regulation of cell cycle and CAM-DR by enhancing p-AKT expression		Ding et al., 2017
Kpnβ 1	Up	Associate with the proliferation, interact with p65, and promote CAM-DR	NF-κB	He et al., 2016
RBQ3 ARF1 VPS4B PKM2 GPR37 RUNX2	Down	Promote proliferation, knockdown of molecules induced CAM-DR	PI3K/Akt MAPK/ERK	Huang et al., 2014; He Y. et al., 2015; Tang et al., 2015; Liu et al., 2016; Xu et al., 2017; Zhang et al., 2020
Homer1b/c	Down	Pro-apoptotic		Tang et al., 2016

#### Non-hodgkin’s Lymphoma (NHL)

Non-Hodgkin’s lymphoma is a group of malignant tumors originating from lymph nodes and extranodal lymphoid tissues, accounting for more than 90% of all lymphomas, including B-cell and NK/T-cell lymphomas. Due to the high heterogeneity of lymphoma, there are more than 60 subtypes of lymphoma, ranging from low-grade malignancy to highly invasive (Li Y. et al., 2015). The malignant degree of T-cell lymphoma is more aggressive than B-NHL, and the treatment lacks specificity. The clinical diagnosis and treatment of B-NHL strictly follow the standard clinical guidelines, but unfortunately, the wide use of monoclonal antibodies has not significantly improved the prognosis of NHL (Lwin et al., 2007b). Based on the recent advances into the tumor microenvironment of NHL, it revealed that CAM-DR is also a crucial factor leading to the recurrence and death (Burger et al., 2009). Studies confirmed that lymphoma cells adhere to BMSCs in the tumor microenvironment to produce a multi-drug resistance (MDR) phenotype, and interrupting this cell adhesion-mediated signal can significantly enhance the sensitivity of tumor cells to chemotherapeutic drugs. When the normal adhesion between malignant tumor cells and the ECM is lost, tumor cells are prone to apoptosis, which is called “anoikis” (Alderton, 2015).

In the related researches of CAM-DR in NHLs, the expressions and functions of characteristic molecules were similar. Despite the common pathological types, there are rare pathological types involving Burkitt’s lymphoma (BL) and mantle cell lymphoma (MCL). Adhesion molecules such as surface chemokine receptors CXCR4, CXCR5, and CXCR7 (Kurtova et al., 2009; Xargay-Torrent et al., 2013) and integrin family molecule Integrin α4β1 (Takeda et al., 2020) make tumor cells home and stay in matrix niches to escape the killing effect of drugs. Eke and Cordes (2015) found that the signal cascade mediated by integrins and the changes of cell cycle and apoptosis are the main mechanisms of CAM-DR production. The anti-CD49d monoclonal antibody or polypeptide that competitively binds with Integrinα4/CD49d ligand can inhibit the adhesion of malignant B-NHL cells to stromal cells and enhance the activity of cytotoxic drugs (Takeda et al., 2020).

Due to the diversity of pathological types in lymphomas, the interactions between abnormally expressed molecules are also complex, which also play cross roles in the biological behaviors of NHLs. In particular, they have different regulations on the proliferation of tumor cells. The expression of some molecules can promote the proliferation of tumor cells, and at the same time, CAM-DR is a positive factor of poor prognoses, such as ADAM12, FBP1 (Huang et al., 2016; Yin et al., 2017). Although the expression of some molecules can reduce the proliferation of tumor cells, the production of CAM-DR is consistent with the clinical practice that is not sensitive to the treatment of inert lymphomas, such as DYRK2 (Wang et al., 2015). Some molecules express the proliferation of tumor cells while producing CAM-DR, which is consistent with the clinical practice that is not sensitive to the treatment of indolent lymphomas, such as DYRK2. Some molecules can reverse CAM-DR and promote proliferation, which is the research direction of clinical targeted therapeutic intervention, such as CKIP-1 and SGTA (Wang et al., 2014; Zhu et al., 2017). The relevant summary is shown in Table 2.

**TABLE 2:** CAM-DR in NHL.

Tumor type	Molecules	Expression level	Biological functions	Signaling pathways	References
MCL	SOX11	Up	Increase migration, transmigration, proliferation, and resistance	FAK/PI3K/AKT p38/MAPK	Balsas et al., 2017; Yang et al., 2020
MCL	CXCL12/CXCR7	Up	Enhance CAM-DR and migration		Kurtova et al., 2009; Xargay-Torrent et al., 2013
BL	CD49D/E	Up	Enhance CAM-DR	NF-κB	Takeda et al., 2020
DLBCL	HGF/MET	Up	Induce integrin-mediated adhesion	RAS/MAPK PI3K/PKB	Tjin et al., 2006
DLBCL	ABCG2	Up	Increase expression of the antiapoptotic proteins		Singh et al., 2011
B-NHL	Integrin α4β1	Up	CAM-DR and inhibit cell apoptosis		Mraz et al., 2011
B-NHL	PRMD1 topoisomerase IIβMDR1	Up	Enhance CAM-DR		Hazlehurst et al., 2006; Lin et al., 2011; Yagi et al., 2013
B-NHL	BAFF hPEBP4	Up	Anti-apoptotic		Lwin et al., 2009; Wang et al., 2013
NHL	p27	Up	Regulate cell-cycle arrest		Lwin et al., 2007a
NHL	XIAP	Up	Anti-apoptotic	NF-κB	Lwin et al., 2007b
NHL	HRF/TCTP	Up	Anti-apoptotic and enhance CAM-DR		He S. et al., 2015
NHL	microRNA-181a	Up	Enhance CAM-DR		Lwin et al., 2010
NHL	TRIP6 CKIP-1 SGTA	Down	Promote proliferation, knockdown of molecules induced CAM-DR	PI3K/AKT	Wang et al., 2014; Miao et al., 2016b; Zhu et al., 2017
NHL	DIXDC1 YB-1 ENO1 Sam68 ADAM12 FBP1	Up	Promote proliferation and CAM-DR	PI3K/AKT	Wu et al., 2015; Zhu X. et al., 2015; Huang et al., 2016; Miao et al., 2016a; Ouyang et al., 2016; Yin et al., 2017
NHL	DYRK2	Up	Decrease proliferation but enhance CAM-DR		Wang et al., 2015

#### Leukemia

Leukemia is a disease characterized by abnormal tumor proliferation of cells in the hematopoietic system and extensive infiltration of extramedullary organs, and abnormal proliferation, differentiation, and apoptosis of peripheral blood leukocytes. It can be divided into different disease types according to the course of the disease, cell morphology, chromosome abnormality, and so on. The specific leukemia subtypes of interest were acute lymphocytic leukemia (ALL), chronic lymphocytic leukemia (CLL), acute myeloid leukemia (AML), and CML (Li et al., 2021). Chemotherapy remains the main treatment of leukemia. With the development of stem cell transplantation technology in recent years, the treatment and prognosis of leukemia have been improved significantly. However, chemotherapy resistance is still a problem that cannot be ignored, which directly leads to insensitivity to first-line treatment or recurrence after short-term remission. The classic drugs for the treatment of leukemia are cell-cycle non-specific killing drugs, which do not have the characteristics of targeted killing of tumor cells. Although the research of targeted drugs is continuously deepening, however, there is still no breakthrough in disease control and prognosis. In contrast, the molecular interaction between the microenvironment and leukemic cells is often ignored (Ruan et al., 2020). Similar to previous myeloma and lymphoma, the tumor microenvironment plays a key role in the treatment of leukemia (Wang et al., 2018; Heath et al., 2019).

Chemotherapeutic resistance in adults with ALL is still an insurmountable problem (Roboz et al., 2014). Although the survival rates of childhood ALL are improving, the recurrence of disease after chemotherapy is still unsolved (Bhojwani et al., 2015). El Azreq et al. (2012) report that collagen/β1 integrin signaling inhibits doxorubicin-induced apoptosis of leukemic T -cells by up-regulating the expression of the ATP-binding cassette C 1 (ABCC1) transporter. Their results indicate for the first time that collagen/β1 integrin/ERK signaling activation could represent a key pathway in T-ALL chemoresistance. In CLL, it has been reported that CAM-DR can activate the phosphatidylinositol 3-kinase (PI3K)/AKT signal pathway, which in turn leads to proliferation (Hoellenriegel et al., 2011). *In vitro* experiments showed that PI3/Akt signaling pathways were related to poor prognosis and drug resistance, and the reduction of apoptosis caused by regulatory chemotherapy in pediatric pre-B ALL (Morishita et al., 2012). In CLL and AML, inhibition of the PI3K/AKT pathway can lead to a decrease in cell proliferation (Billottet et al., 2006; Nguyen et al., 2014). Among the studies of CAM-DR in the hematological tumors mentioned above, it has been found that PI3K/AKT is involved in the occurrence and development of CAM-DR in MM and NHL (Ouyang et al., 2016; Huang et al., 2019). Moreover, in the solid tumor, adhesion-mediated activation of the PI3K/AKT pathway has also been reported (Zhu et al., 2012; Toth et al., 2019). Due to the relations between PI3K activation and the unlimited proliferation of tumor cells and CAM-DR,PI3K/AKT has become a promising target in anti-tumor therapies. As part of the combined treatment, the PI3K inhibitors can not only inhibit tumor cell proliferation but also restore sensitivity to other treatments, reduce the occurrence of drug resistance and achieve a synergistic killing effect. Further studies on PI3K/AKT signaling pathway inhibitors have found that inhibition of related signaling pathways can significantly inhibit tumor cell proliferation and enhance chemosensitivity in CLL and AML (Billottet et al., 2006; Nguyen et al., 2014). Some results showed that by inhibiting pro-apoptotic Ras-related C3 botulinum toxin substrate 1 (Rac1), α2β1 integrin can be a major pathway protecting leukemic cells from genotoxic agents and may thus represent an important therapeutic target in the anti-cancer treatment of AML (Naci et al., 2019). Bruton tyrosine kinase (BTK) inhibitors have also been found to reverse CAM-DR and tumor migration in CML (de Rooij et al., 2012). Fernandez-Vidal et al. (2006) have shown that the inhibition of proliferation and anti-apoptosis induced by adhesion is related to the expression of M-phase inducer phosphatase 1 (CDC25A), while CDC25A participates in the regulation of the cell cycle and activates the PI3K/AKT signal pathway. CDC25A is a bispecific phosphatase, which mainly participates in the G1-S transition of the cell cycle, and plays an important role in the regulation of the cell cycle involving CAM-DR. Previous shreds of evidence suggested that integrins play key roles in the CAM-DR of MM and NHL, and existing studies also indicated that integrin α4 plays a similar role in leukemia’s drug resistance (Shishido et al., 2014). Incomplete sensitivity to chemotherapy leads to the persistence of some drug-resistant cells and minimal residual diseases (MRD) (Akabane and Logan, 2020). Interfering integrin α4-mediated cell adhesion can make them sensitive to chemotherapy, thus further promoting the killing effects of all tumor cells in an MRD setting.

### Solid Tumors

The incidence of hepatocellular carcinoma (HCC) is high in Asia. Only about 20% of patients have the chance of surgical resection, and most patients have a short-term risk of recurrence after surgery (Saito et al., 2019). Despite the development of interventional therapy, targeted therapy, and immunotherapy, the overall prognosis of HCC is still poor and it remains the second leading cause of cancer-related deaths in the world (Ferlay et al., 2015). Moreover, most HCCs are resistant to chemotherapeutic drugs (Wu et al., 2021). With the continuous study of the tumor microenvironment, it is found that CAM-DR in HCC is similar to hematological tumors. In previous studies, it has been found that the adhesion of HepG2 cells to FN can significantly increase the drug resistance of tumor cells, and is closely related to the expression of integrin β1 (Zhu et al., 2012). At the same time, some studies have shown that the regulation of connective tissue growth factor (CTGF) and collagen 1A1 (COL1A1) is involved in CAM-DR (Song et al., 2017).

In addition to HHC, CAM-DR is also being investigated in many epithelial tumors. CAM-DR can occur in almost all kinds of tumors. Nakagawa et al. (2014) found that the involvement of integrin β1 in the adhesion of tumor cells to FN can lead to 5-Fu resistance in the study of oral squamous cell carcinoma (OSCC). In head and neck cancer, a melphalan-resistant nasopharyngeal carcinoma (NPC) cell line shows up-regulation of integrin subunits α2, α5, α6, β1, and β2 compared with drug-sensitive parent lines. As well as chemoresistance phenotype, this was associated with a significantly stronger binding to ECM and increased invasiveness (Liang et al., 2001). A vinblastine-resistant subline of renal carcinoma cells showed increased expression of integrin very late antigen-1/2 and decreased expression of integrin very late antigen-6 in association with increased attachment to collagen and FN (Duensing et al., 1996). Similar results have been found in gastric cancer (GC). MGr1-Ag/37LRP (P37-kDa laminin receptor precursor) can promote CAM-DR by activating downstream focal adhesion kinase (FAK)/PI3K and mitogen-activated protein kinase (MAPK) signal pathways through interaction with laminin (Sun et al., 2014b). Common intracellular signals related to integrin binding primarily start with the activation of FAK, recruitment, and activation of Src kinase family, followed by PI3K/Akt pathway or Ras/RAF/MEK/ERK signaling axis, resulting in increased invasion and survival (Li W. et al., 2015; Hou et al., 2016). The mechanistic impact of integrin on chemosensitivity showed high diversity, and a significant increase in MAPK and CREB signal pathways could be found in CAM-DR (Jakubzig et al., 2018). In other words, integrin-mediated matrix binding induces crosstalk with the growth factor signal axis, such as EGFR via FAK or MAPK/ERK pathways triggering proliferation and reducing apoptosis (Jeanes et al., 2012). Although the mechanism of the MAPK/ERK signaling pathway is still relatively thorough compared with the above two pathways, the research of corresponding inhibitors is also relatively small, but it is also believed to be a research direction of the targeted treatment of drug resistance in the future. When human breast cancer cell lines MCF-7 and MDA-MB-231 bind to collagen type 1 (COL1) or FN, they are less sensitive to the cytotoxicity of cisplatin, doxorubicin, and mitoxantrone. CAM-DR is also closely related to the regulation of integrin β1 in breast cancer (Baltes et al., 2020). The expression of integrin β1 also plays an important role in CAM-DR and is considered to be a potential target for non-small cell lung cancer (NSCLC) (Wang et al., 2020). Adhesion of small-cell lung cancer (SCLC) cells to ECM enhances tumorigenicity and confers resistance to chemotherapeutic agents as a result of β1 integrin-stimulated tyrosine kinase activation suppressing chemotherapy-induced apoptosis (Sethi et al., 1999). Strategies based on blocking β1 integrin-mediated survival signals may represent a new therapeutic approach to improve the response to chemotherapy in SCLC (Rintoul and Sethi, 2001). In some special types of tumors, researches on CAM-DR have also been conducted, such as glioblastoma multiforme (GBM) and glioma, blocking the interaction between tumor cells and microenvironment can reverse the drug resistance (Westhoff et al., 2008; Ding et al., 2020).

In human reproductive system tumors, hormone receptor-dependent diseases can be treated with endocrine drugs and/or potential treatments. In reproductive system-related tumors, the primary and acquired drug resistance of endocrine and chemotherapeutic drugs have become the hotspots of current researches (Buttigliero et al., 2015; Lin et al., 2019). In bone-metastatic castration-resistant prostate cancer (CRPC), the interaction between tumor cells and microenvironment combined with hypoxia can lead to the continuous activation of the PI3K signaling pathway, which eventually leads to drug resistance. CAM-DR is mainly regulated by Integrin α6β1, while hypoxia is related to the regulation of PIM kinase in CRPC (Toth et al., 2019). In ovarian cancer, the expression of Lewis *y* is significantly high, and it is positively correlated with the expression of some adhesion molecules. The adhesion molecules integrin α5 and integrin β1 can be used as independent prognostic factors to judge progression-free survival (PFS) and overall survival (OS) in ovarian cancer (Zhu L.C. et al., 2015). Lewis *y* inhibits apoptosis and increases CAM-DR in ovarian cancer mainly by activating the FAK signal pathway and inhibiting the BCL-2/BCL-XL pathway (Yan et al., 2015). The above studies on CAM-DR in solid tumors are summarized in Table 3.

**TABLE 3:** Roles and mechanisms of CAM-DR in solid tumors.

Tumor type	Molecules	Biological functions	Signaling pathways	References
OSCC	FN	Enhance chemosensitivity to 5-FU and apoptosis	ILK/Akt/NF-κB	Nakagawa et al., 2014
NPC	Integrin subunits α2, α5, α6, β1, and β2	Chemoresistance phenotype and increase invasiveness		Liang et al., 2001
HCC	CTGF/COL1A1	Form of compact spheroids and evade anticancer therapies		Song et al., 2017
HCC	Integrinβ1	Decrease apoptosis	PI3K/AKT	Zhu et al., 2012
Bone-metastatic CRPC	Integrin α6β1/PIM	Promote survival by reducing oxidative stress and preventing cell death	PI3K/mTOR	Toth et al., 2019
GC	MGr1-Ag/37LRP	Ligation-induced adhesion participated in protecting cells from some apoptotic stimuli caused by chemotherapeutic drugs	FAK/PI3K and ERK/MAPK	Liu et al., 2010; Sun et al., 2014a,b
Ovarian cancer	Lewis *y*	Enhance CAM-DR	FAK	Yan et al., 2015; Zhu L.C. et al., 2015
Breast cancer	Integrinβ1	Transmits breast cancer cells into chemoresistance	ERK/MAPK	Baltes et al., 2020
Breast cancer	Integrinβ1	Inhibits drug-induced apoptosis by inhibiting the release of cytochrome c from the mitochondria	PI3K/AKT	Aoudjit and Vuori, 2001
NSCLC	Integrinβ1	Increase cell viability, promote migration and adhesion		Wang et al., 2020
SCLC	Integrinβ1	G2/M cell cycle arrest by up-regulate p21^*Cip1/WAF1*^ and p27^*Kip1*^ and the down-regulate of cyclins E, A, and B; prevents etoposide-induced caspase-3 activation and subsequent apoptosis	PI3K/AKT	Hodkinson et al., 2006, 2007
GMB		Mode of CAM-DR by forming spheres via cell–cell interactions	Raf/MEK/ERK and PI3K/AKT	Westhoff et al., 2008
Glioma	β-catenin	Enhance cell adhesion contributed to the insensitivity to Temozolomide	IP3R1/AKT/β-catenin	Ding et al., 2020

## Therapeutic Drugs and Clinical Application

### Inhibitors

With the deepening of CAM-DR studies, there are more and more studies on inhibitors. Reversing CAM-DR can significantly improve drug resistance, reduce recurrence, improve the prognosis of patients, and provide a leap forward in clinical treatment. At present, there are some difficulties in targeting molecules. Although there are significant differences in expression, they lack specificity. Some monoclonal antibodies have been proven to be effective in reversing CAM-DR, and the combined use of traditional Chinese medicine can often inhibit the CAM-DR phenomenon. Most studies and clinical applications are related to signal pathway inhibitors (see Table 4 for details).

**TABLE 4:** Overview of the targeting drugs.

Classification	Name	Disease	Target	Mechanism	Phase	References
CXCR4 inhibitors	Plerixafor (ADM3100) BKT140	MM and MCL	CXCR4/CXCL12	CXCR4 antagonist; abrogate CXCL12 induced receptor internalization; induce MM cell apoptosis	Clinical trial	Kurtova et al., 2009; Balsas et al., 2017; Waldschmidt et al., 2017; Wang et al., 2019
Anti-integrin antibody	Natalizumab	MM, MCL, and B-NHL	Integrin α4β1/7	Non-competitive antagonism	FDA approve	Kobune et al., 2007; Mraz et al., 2011; Xargay-Torrent et al., 2013
	E7820 GBR-500	HCC	Integrin α2	Block the local growth and increases the sensitivity of cancer cell lines to cytotoxic drugs	Phase II clinical trials	Keizer et al., 2011; Mita et al., 2011; Naci et al., 2015
VLA4-nanoparticles	V-NP/V-CP	MM	Integrinα 4β1	V-CP: anti-myeloma effects. V-CP: further reduced tumor burden and prolonged survival without adding to toxicity.	*In vitro*	Fontana et al., 2021
Selectin inhibitors	Uproleselan (GMI-1271)	AML	E-selectin	Small molecule inhibitor against E-selectin on endothelial cells	*In vitro*	Barbier et al., 2020
PI3K inhibitors	Idelalisib Copansilib Duvelisib Alpelisib (BYL719)	Leukemia breast cancer	PI3K	Inhibit PI3K/AKT signaling pathway	FDA approve	Miller et al., 2015; Flinn et al., 2018a,b; Mensah et al., 2018
	Buparlisib (BKM120) Dactolisib (BEZ235)	Leukemia	PI3K	Inhibit PI3K/AKT signaling pathway	Phase I clinical trials	Ragon et al., 2017; Stefanzl et al., 2017
	LY294002	MM, NHL, bone-metastatic CRPC, SCLC	PI3K	Inhibit PI3K/AKT signaling pathway	*In vitro*	Aoudjit and Vuori, 2001; Xu et al., 2017; Huang et al., 2019; Toth et al., 2019
AKT inhibitors	MK2206 PF-04691502 A6730	MM and NHL	AKT	Inhibit PI3K/AKT signaling pathway	*In vitro*	Wu et al., 2015; Ding et al., 2017; Zhang et al., 2020
MAPK inhibitor	PD98059 SB203580	MM and MCL	MAPK	ERK/MAPK signaling pathway inhibitors	*In vitro*	Balsas et al., 2017; Xu et al., 2017; Yang et al., 2020
ABCG2/BRCP inhibitor	Fumitremorgin	DLBCL	ABCG2/BRCP	Abrogate the stroma-induced chemotolerance	*In vitro*	Singh et al., 2011
Cyclopamine derivative	Cyclopamine-KAAD	DLBCL	Hedgehog signaling	Inhibit hedgehog signaling	*In vitro*	Singh et al., 2011
Nanomedicine	PDGFR-β - doxorubicin	Tumors	Hedgehog signaling	Increase the binding of doxorubicin and reduce the free doxorubicin	*In vitro*	Prakash et al., 2010
Others	FNIII14	OSCC	FN	Enhance chemosensitivity to 5-FU and apoptosis	*In vitro*	Nakagawa et al., 2014
	Losartan	HCC	CTGF/COL1A1	Decrease the form of compact spheroids	*In vitro*	Song et al., 2017
	Oroxylin A	HCC, glioma	β-catenin	inhibit IP3R1/AKT/β-catenin pathway	*In vitro*	Zhu et al., 2012; Ding et al., 2020
	Wenxia Changfu formula (WCF)	NSCLC	Integrinβ1	Decrease cell adhesion	*In vitro*	Wang et al., 2020
	Carbenoxolone	GMB		Sensitize to CD95-induced apoptosis	*In vitro*	Westhoff et al., 2008

It has been made clear that the occurrence of CAM-DR is closely related to the activation of related signal pathways, and there are more and more studies on inhibitors of signal pathways. More sophisticated inhibitors of the PI3K/AKT signaling pathway compared to others. In the *in vitro* experiment, the addition of PI3K inhibitor can significantly reduce the proliferation of tumor cells, reduce the adhesion of tumor cells to the microenvironment, and reverse drug resistance. In the treatment of hematological malignant tumors, the research of PI3K inhibitors presents different stages. The inhibitors approved by FDA are idelalisib, copansilib, and duvelisib. Idelalisib, also known as CAL101, was approved for inert non-Hodgkin’s Lymphoma (iNHL) in July 2014 (Miller et al., 2015). The approval is based on the results of a single-arm phase II study in which the overall remission rate (ORR) of idelalisib in patients with follicular lymphoma (FL) and small lymphocytic lymphoma (SLL) was 54 and 58%, respectively (Furman et al., 2014; Gilbert, 2014). Copanlisib is the second PI3K inhibitor approved for recurrent FL patients by the FDA in September 2017 (Mensah et al., 2018; Tang et al., 2018). Compared with Idelalisib, the high affinity for the p110 subunit of PI3K makes it provide higher specificity and lower gastrointestinal toxicity (Krause et al., 2018). Clinical trials of copanlisib are performed in multiple subtypes of NHL in order to further expand its clinical indications. Duvelisib (also known as ABBV-954, INK-1197, and IPI-145) is an oral PI3K inhibitor for the treatment of hematological malignant tumors (Flinn et al., 2018a,b). In September 2018, FDA approved duvelisib for more than two lines of treatment for patients with recurrent/refractory FL and CLL/SLL (Blair, 2018; No author list, 2018). The approval of the FDA is based on the improvement of survival and treatment efficacy of phase III DUO and phase II DYNAMO trials. In the DUO study, the median PFS of CLL/SLL patients receiving drug treatment was 16.4 months and ORR was 78%. In contrast, patients who received CD20 monoclonal antibodies had a median PFS of 9.1 months and an ORR of 39% (Flinn et al., 2018a). Currently, the researchers are dedicating to develop more PI3K pathway inhibitors, and some inhibitors are also in preliminary clinical trials or *in vitro* experiments, such as Buparlisib (BKM120) and Dactolisib (BEZ235) (Ragon et al., 2017; Stefanzl et al., 2017). While improving the curative effect, we should also pay more attention to the side effects caused by the inhibitors.

Compared with PI3K inhibitors, NF-κB pathway inhibitors have been widely used in MM, such as bortezomib (BTZ), which induces apoptosis by inhibiting the degradation of IκBα and inhibiting the activation of NF-κB in cancer cells (Murray and Norbury, 2000). As a small molecule NF-κB signal inhibitor, V1810 directly inhibits NF-κB by non-relying on the proteasome mechanism to induce apoptosis of MM cells (Kurland et al., 2001). P38 MAPK inhibitor SB203580 can inhibit tumor microenvironment-induced proliferation in MCL *in vitro* (Yang et al., 2020). Although FAK inhibitor (PF) only reduces cell chemotaxis and *trans*-endothelial migration, the potential ability to prevent tumor cells from bone marrow and lymph node microenvironment protection by recirculation makes it a synergistic drug of AnticD20 or BTZ (Balsas et al., 2017). Strategies designed to target down-stream of cell adhesion molecules such as FAK or integrin linked kinase may eliminate concerns of redundancy of adhesion-mediated signaling. Similarly, inhibiting the Janus-activated kinase (JAK)/signal transduction and activator of transcription (STAT) pathway for cytokine signaling maybe more effective comparing to the blockade of a single cytokine receptor (Chen et al., 2020).

### Nanomedicine Therapeutic

Nanomedicine is an emerging form of treatment, which focuses on the delivery of alternative drugs and improvement of therapeutic effects, while reducing harmful side effects on normal tissues. The novel nanomedicines based on the tumor genetic spectrum can design and produce drugs flexibly and quickly, which makes the drug selection of personal treatment more centralized and effective (Khot et al., 2021). With the advanced design and alternative drug delivery mechanisms of different nanodrugs (including liposomes, dendrimers, micelles, carbon-based, polymer conjugates, and metal nanoparticles), overcoming various forms of multidrug resistance looks promising and opens up a new field of vision for cancer treatment (Markman et al., 2013). For example, a unique nano-carrier was made using albumin and a platelet-derived growth factor receptor-β (PDGFR-β) recognizing cyclin peptide conjugated to doxorubicin through an acid-sensitive hydrazone linkage. *In vivo*, the binding of doxorubicin can be increased, and the free doxorubicin can be reduced, thereby reducing the expression of PDGFR-β, significantly reducing tumor growth (Prakash et al., 2010). A large number of unique nanodrugs have been created and widely studied, and have entered the stage of clinical development. With more discoveries and drug optimization, the advantages of nanodrugs over current treatment options will continue to be enhanced, thus effectively eradicate drug-resistant cancers.

## Conclusion and Prospects

In the continuous improvement of tumor clinical treatment, drug resistance has become a non-negligible hotspot, which is a key factor for successful treatment. With the advancement and improvement of clinical treatment technology, overcoming drug resistance has become a key issue to improve treatment effect and prognosis of patients. CAM-DR is an important factor in drug resistance caused by the tumor microenvironment. A large number of studies are focusing on CAM-DR, covering hematologic malignant tumors to solid tumors, and the mechanisms insight are gradually being revealed. According to these mechanisms, the study of corresponding inhibitors and combined therapies can reverse the occurrence of drug resistance to a great extent. However, the mechanism of CAM-DR is very complex, involving the activation of multiple signal pathways, and the development of corresponding inhibitors and clinical trials are also underway, which is expected to improve the existing treatment to a higher level in the future. With the deepening of CAM-DR research, we believe that overcoming CAM-DR can provide a new method for tumor treatment. Taken together, previous studies demonstrate that CAM-DR is crucial in the drug resistance mechanisms and reversing CAM-DR might provide a promising therapeutic strategy for clinicians.

## Author Contributions

YH and YW collected the related article and finished the manuscript and figures. SJ and SH gave constructive guidance and made critical revisions. XS participated in the design of this review. JT and SQ revised the manuscript. All authors read and approved the final manuscript.

## Conflict of Interest

The authors declare that the research was conducted in the absence of any commercial or financial relationships that could be construed as a potential conflict of interest.

## Abbreviations

EM-DR: environment-mediated drug resistance
SM-DR: soluble factor mediated-drug resistance
CAM-DR: cell adhesion mediated-drug resistance
NHL: non-Hodgkin’s lymphoma
MM: multiple myeloma
VEGF: vascular growth factor
IL-3, IL-6: interleukin-3, 6
NO: nitric oxide
G-CSF: granulocyte colony-stimulating factor
BAFF: B cell-activating factor of the TNF family
GM-CSF: granulocyte-macrophage colony-stimulating factor
Bcl-2: B cell lymphoma-2
FN: fibronectin
VCAM-1: vascular cell adhesion molecule-1
VLA-4: integrin very late antigen-4
DDR: discoidin domain receptor
CXCR: C-X -C motif chemokine receptors
BMSCs: bone marrow stromal cells
MGUS: monoclonal gammopathy of unknown significance
SMM: smoldering multiple myeloma
HDACs: histone deacetylases
CAFs: cancer-associated fibroblasts
MDR: multi-drug resistance
BL: Burkitt’s lymphoma
MCL: mantle cell lymphoma
ALL: acute lymphocytic leukemia
CLL: chronic lymphocytic leukemia
AML: acute myeloid leukemia
CML: chronic myeloid leukemia
ABCC1: ATP-binding cassette C 1
PI3K: phosphatidylinositol 3-kinase
BTK: Bruton tyrosine kinase
CDC25A: M-phase inducer phosphatase 1
MRD: minimal residual diseases
HCC: hepatocellular carcinoma
CTGF: connective tissue growth factor
COL1A1: collagen 1A1
OSCC: oral squamous cell carcinoma
NPC: nasopharyngeal carcinoma
ECM: extracellular matrix
GC: gastric cancer
FAK: focal adhesion kinase
MAPK: mitogen-activated protein kinase
COL1: collagen type 1
NSCLC: non-small cell lung cancer
SCLC: small-cell lung cancer
GBM: glioblastoma multiforme
CRPC: castration-resistant prostate cancer
PFS: progression-free survival
OS: overall survival
JAK: Janus-activated kinase
STAT: signal transduction and activator of transcription
iNHL: inert non-Hodgkin’s Lymphoma
ORR: overall remission rate
SLL: small lymphocytic lymphoma
FL: follicular lymphoma
BTZ: bortezomib
PDGFR- β: platelet-derived growth factor receptor- β.

## References

[B1] Akabane H. Logan A. (2020). Clinical significance and management of MRD in adults with acute lymphoblastic leukemia. *Clin. Adv. Hematol. Oncol.* 18 413–422. 32903253

[B2] Alderton G. K. (2015). Therapeutic resistance: fibroblasts restrain drug sensitivity. *Nat. Rev. Cancer* 15 318–319. 10.1038/nrc3965 25998706

[B3] Aoudjit F. Vuori K. (2001). Integrin signaling inhibits paclitaxel-induced apoptosis in breast cancer cells. *Oncogene* 20 4995–5004. 10.1038/sj.onc.1204554 11526484

[B4] Balsas P. Palomero J. Eguileor A. Rodriguez M. L. Vegliante M. C. Planas-Rigol E. (2017). SOX11 promotes tumor protective microenvironment interactions through CXCR4 and FAK regulation in mantle cell lymphoma. *Blood* 130 501–513. 10.1182/blood-2017-04-776740 28533307

[B5] Baltes F. Pfeifer V. Silbermann K. Caspers J. Wantoch von Rekowski K. Schlesinger M. (2020). beta1-Integrin binding to collagen type 1 transmits breast cancer cells into chemoresistance by activating ABC efflux transporters. *Biochim. Biophys. Acta Mol. Cell Res.* 1867:118663. 10.1016/j.bbamcr.2020.118663 31987794

[B6] Barbier V. Erbani J. Fiveash C. Davies J. M. Tay J. Tallack M. R. (2020). Endothelial E-selectin inhibition improves acute myeloid leukaemia therapy by disrupting vascular niche-mediated chemoresistance. *Nat. Commun.* 11:2042. 10.1038/s41467-020-15817-5 32341362 PMC7184728

[B7] Bhojwani D. Yang J. J. Pui C. H. (2015). Biology of childhood acute lymphoblastic leukemia. *Pediatr. Clin. North Am.* 62 47–60. 10.1016/j.pcl.2014.09.004 25435111 PMC4250840

[B8] Billottet C. Grandage V. L. Gale R. E. Quattropani A. Rommel C. Vanhaesebroeck B. (2006). A selective inhibitor of the p110delta isoform of PI 3-kinase inhibits AML cell proliferation and survival and increases the cytotoxic effects of VP16. *Oncogene* 25 6648–6659. 10.1038/sj.onc.1209670 16702948

[B9] Bistrian R. Dorn A. Mobest D. C. Ruster B. Ludwig R. Scheele J. (2009). Shear stress-mediated adhesion of acute myeloid leukemia and KG-1 cells to endothelial cells involves functional P-selectin. *Stem Cells Dev.* 18 1235–1242. 10.1089/scd.2008.0380 19105599

[B10] Blair H. A. (2018). Duvelisib: first global approval. *Drugs* 78 1847–1853. 10.1007/s40265-018-1013-4 30430368

[B11] Borsig L. (2018). Selectins in cancer immunity. *Glycobiology* 28 648–655. 10.1093/glycob/cwx105 29272415 PMC6711759

[B12] Brito J. L. Walker B. Jenner M. Dickens N. J. Brown N. J. Ross F. M. (2009). MMSET deregulation affects cell cycle progression and adhesion regulons in t(4;14) myeloma plasma cells. *Haematologica* 94 78–86. 10.3324/haematol.13426 19059936 PMC2625417

[B13] Buck E. Gokhale P. C. Koujak S. Brown E. Eyzaguirre A. Tao N. (2010). Compensatory insulin receptor (IR) activation on inhibition of insulin-like growth factor-1 receptor (IGF-1R): rationale for cotargeting IGF-1R and IR in cancer. *Mol. Cancer Ther.* 9 2652–2664. 10.1158/1535-7163.MCT-10-0318 20924128

[B14] Burger J. A. Ghia P. Rosenwald A. Caligaris-Cappio F. (2009). The microenvironment in mature B-cell malignancies: a target for new treatment strategies. *Blood* 114 3367–3375. 10.1182/blood-2009-06-225326 19636060 PMC4969052

[B15] Buttigliero C. Tucci M. Bertaglia V. Vignani F. Bironzo P. Di Maio M. (2015). Understanding and overcoming the mechanisms of primary and acquired resistance to abiraterone and enzalutamide in castration resistant prostate cancer. *Cancer Treat. Rev.* 41 884–892. 10.1016/j.ctrv.2015.08.002 26342718

[B16] Chen C. Zhang H. X. Wang M. Song X. G. Cao J. Wang L. (2014). Stromal cells attenuate the cytotoxicity of imatinib on Philadelphia chromosome-positive leukemia cells by up-regulating the VE-cadherin/beta-catenin signal. *Leuk. Res.* 38 1460–1468. 10.1016/j.leukres.2014.09.012 25443888

[B17] Chen W. C. Hu G. Hazlehurst L. A. (2020). Contribution of the bone marrow stromal cells in mediating drug resistance in hematopoietic tumors. *Curr. Opin. Pharmacol.* 54 36–43. 10.1016/j.coph.2020.08.006 32898723 PMC7770000

[B18] Dalton W. S. (2003). The tumor microenvironment: focus on myeloma. *Cancer Treat. Rev.* 29(Suppl. 1), 11–19. 10.1016/s0305-7372(03)00077-x 12738239

[B19] Damiano J. S. Cress A. E. Hazlehurst L. A. Shtil A. A. Dalton W. S. (1999). Cell adhesion mediated drug resistance (CAM-DR): role of integrins and resistance to apoptosis in human myeloma cell lines. *Blood* 93 1658–1667. 10029595 PMC5550098

[B20] de Rooij M. F. Kuil A. Geest C. R. Eldering E. Chang B. Y. Buggy J. J. (2012). The clinically active BTK inhibitor PCI-32765 targets B-cell receptor- and chemokine-controlled adhesion and migration in chronic lymphocytic leukemia. *Blood* 119 2590–2594. 10.1182/blood-2011-11-390989 22279054

[B21] Dehghanifard A. Kaviani S. Abroun S. Mehdizadeh M. Saiedi S. Maali A. (2018). Various signaling pathways in multiple myeloma cells and effects of treatment on these pathways. *Clin. Lymphoma Myeloma Leuk.* 18 311–320. 10.1016/j.clml.2018.03.007 29606369

[B22] Di Marzo L. Desantis V. Solimando A. G. Ruggieri S. Annese T. Nico B. (2016). Microenvironment drug resistance in multiple myeloma: emerging new players. *Oncotarget* 7 60698–60711. 10.18632/oncotarget.10849 27474171 PMC5312413

[B23] Dias S. Choy M. Alitalo K. Rafii S. (2002). Vascular endothelial growth factor (VEGF)-C signaling through FLT-4 (VEGFR-3) mediates leukemic cell proliferation, survival, and resistance to chemotherapy. *Blood* 99 2179–2184. 10.1182/blood.v99.6.2179 11877295

[B24] Ding L. Shen Y. Ni J. Ou Y. Liu H. (2017). EphA4 promotes cell proliferation and cell adhesion-mediated drug resistance via the AKT pathway in multiple myeloma. *Tumour Biol.* 39:1010428317694298. 10.1177/1010428317694298 28351297

[B25] Ding Y. Zhou Y. Li Z. Zhang H. Yang Y. Qin H. (2020). Oroxylin A reversed Fibronectin-induced glioma insensitivity to Temozolomide by suppressing IP3R1/AKT/beta-catenin pathway. *Life Sci.* 260:118411. 10.1016/j.lfs.2020.118411 32918978

[B26] Duensing S. Brevis Nunez F. Meyer N. Anastassiou G. Nasarek A. Grosse J. (1996). Exposure to vinblastine modulates beta 1 integrin expression and in vitro binding to extracellular matrix molecules in a human renal carcinoma cell line. *Invasion Metastasis* 16 65–72. 9030241

[B27] Eke I. Cordes N. (2015). Focal adhesion signaling and therapy resistance in cancer. *Semin. Cancer Biol.* 31 65–75. 10.1016/j.semcancer.2014.07.009 25117005

[B28] El Azreq M. A. Naci D. Aoudjit F. (2012). Collagen/beta1 integrin signaling up-regulates the ABCC1/MRP-1 transporter in an ERK/MAPK-dependent manner. *Mol. Biol. Cell* 23 3473–3484. 10.1091/mbc.E12-02-0132 22787275 PMC3431945

[B29] Elliott T. Sethi T. (2002). Integrins and extracellular matrix: a novel mechanism of multidrug resistance. *Expert Rev. Anticancer Ther.* 2 449–459. 10.1586/14737140.2.4.449 12647988

[B30] Ferlay J. Soerjomataram I. Dikshit R. Eser S. Mathers C. Rebelo M. (2015). Cancer incidence and mortality worldwide: sources, methods and major patterns in GLOBOCAN 2012. *Int. J. Cancer* 136 E359–E386. 10.1002/ijc.29210 25220842

[B31] Fernandez-Vidal A. Ysebaert L. Didier C. Betous R. De Toni F. Prade-Houdellier N. (2006). Cell adhesion regulates CDC25A expression and proliferation in acute myeloid leukemia. *Cancer Res.* 66 7128–7135. 10.1158/0008-5472.CAN-05-2552 16854822

[B32] Flinn I. W. Hillmen P. Montillo M. Nagy Z. Illes A. Etienne G. (2018a). The phase 3 DUO trial: duvelisib vs ofatumumab in relapsed and refractory CLL/SLL. *Blood* 132 2446–2455. 10.1182/blood-2018-05-850461 30287523 PMC6284216

[B33] Flinn I. W. O’Brien S. Kahl B. Patel M. Oki Y. Foss F. F. (2018b). Duvelisib, a novel oral dual inhibitor of PI3K-delta, gamma, is clinically active in advanced hematologic malignancies. *Blood* 131 877–887. 10.1182/blood-2017-05-786566 29191916 PMC6033052

[B34] Fontana F. Scott M. J. Allen J. S. Yang X. Cui G. Pan D. (2021). VLA4-targeted nanoparticles hijack cell adhesion-mediated drug resistance to target refractory myeloma cells and prolong survival. *Clin. Cancer Res.* 27 1974–1986. 10.1158/1078-0432.CCR-20-2839 33355244 PMC8026499

[B35] Fortney J. E. Zhao W. Wenger S. L. Gibson L. F. (2001). Bone marrow stromal cells regulate caspase 3 activity in leukemic cells during chemotherapy. *Leuk. Res.* 25 901–907. 10.1016/S0145-2126(01)00051-0 11532524

[B36] Frassanito M. A. Rao L. Moschetta M. Ria R. Di Marzo L. De Luisi A. (2014). Bone marrow fibroblasts parallel multiple myeloma progression in patients and mice: in vitro and in vivo studies. *Leukemia* 28 904–916. 10.1038/leu.2013.254 23995611

[B37] Furman R. R. Sharman J. P. Coutre S. E. Cheson B. D. Pagel J. M. Hillmen P. (2014). Idelalisib and rituximab in relapsed chronic lymphocytic leukemia. *N. Engl. J. Med.* 370 997–1007. 10.1056/NEJMoa1315226 24450857 PMC4161365

[B38] Furukawa Y. Kikuchi J. (2016). Epigenetic mechanisms of cell adhesion-mediated drug resistance in multiple myeloma. *Int. J. Hematol.* 104 281–292. 10.1007/s12185-016-2048-5 27411688

[B39] Galan-Diez M. Cuesta-Dominguez A. Kousteni S. (2018). The Bone marrow microenvironment in health and myeloid malignancy. *Cold Spring Harb. Perspect. Med.* 8:a031328. 10.1101/cshperspect.a031328 28963115 PMC6027930

[B40] Gao Y. Zhou J. Li J. (2021). Discoidin domain receptors orchestrate cancer progression: a focus on cancer therapies. *Cancer Sci.* 112 962–969. 10.1111/cas.14789 33377205 PMC7935774

[B41] Gilbert J. A. (2014). Idelalisib: targeting PI3Kdelta in B-cell malignancies. *Lancet Oncol.* 15:e108. 10.1016/s1470-2045(14)70052-x 24809089

[B42] Gilmore T. D. (2007). Multiple myeloma: lusting for NF-kappaB. *Cancer Cell* 12 95–97. 10.1016/j.ccr.2007.07.010 17692798

[B43] Hadzijusufovic E. Albrecht-Schgoer K. Huber K. Hoermann G. Grebien F. Eisenwort G. (2017). Nilotinib-induced vasculopathy: identification of vascular endothelial cells as a primary target site. *Leukemia* 31 2388–2397. 10.1038/leu.2017.245 28757617 PMC5669463

[B44] Hazlehurst L. A. Dalton W. S. (2001). Mechanisms associated with cell adhesion mediated drug resistance (CAM-DR) in hematopoietic malignancies. *Cancer Metastasis Rev.* 20 43–50. 10.1023/a:1013156407224 11831646

[B45] Hazlehurst L. A. Argilagos R. F. Emmons M. Boulware D. Beam C. A. Sullivan D. M. (2006). Cell adhesion to fibronectin (CAM-DR) influences acquired mitoxantrone resistance in U937 cells. *Cancer Res.* 66 2338–2345. 10.1158/0008-5472.CAN-05-3256 16489039

[B46] Hazlehurst L. A. Damiano J. S. Buyuksal I. Pledger W. J. Dalton W. S. (2000). Adhesion to fibronectin via beta1 integrins regulates p27kip1 levels and contributes to cell adhesion mediated drug resistance (CAM-DR). *Oncogene* 19 4319–4327. 10.1038/sj.onc.1203782 10980607

[B47] Hazlehurst L. A. Enkemann S. A. Beam C. A. Argilagos R. F. Painter J. Shain K. H. (2003). Genotypic and phenotypic comparisons of de novo and acquired melphalan resistance in an isogenic multiple myeloma cell line model. *Cancer Res.* 63 7900–7906. 14633719

[B48] Hazlehurst L. A. Valkov N. Wisner L. Storey J. A. Boulware D. Sullivan D. M. (2001). Reduction in drug-induced DNA double-strand breaks associated with beta1 integrin-mediated adhesion correlates with drug resistance in U937 cells. *Blood* 98 1897–1903. 10.1182/blood.v98.6.1897 11535527

[B49] He S. Huang Y. Wang Y. Tang J. Song Y. Yu X. (2015). Histamine-releasing factor/translationally controlled tumor protein plays a role in induced cell adhesion, apoptosis resistance and chemoresistance in non-Hodgkin lymphomas. *Leuk. Lymphoma* 56 2153–2161. 10.3109/10428194.2014.981173 25363345

[B50] He S. Miao X. Wu Y. Zhu X. Yin H. He Y. (2016). Upregulation of nuclear transporter, Kpnbeta1, contributes to accelerated cell proliferation- and cell adhesion-mediated drug resistance (CAM-DR) in diffuse large B-cell lymphoma. *J. Cancer Res. Clin. Oncol.* 142 561–572. 10.1007/s00432-015-2057-4 26498772 PMC11819056

[B51] He Y. Wang Y. Liu H. Xu X. He S. Tang J. (2015). Pyruvate kinase isoform M2 (PKM2) participates in multiple myeloma cell proliferation, adhesion and chemoresistance. *Leuk. Res.* 39 1428–1436. 10.1016/j.leukres.2015.09.019 26453405

[B52] Heath J. L. Cohn G. M. Zaidi S. K. Stein G. S. (2019). The role of cell adhesion in hematopoiesis and leukemogenesis. *J. Cell Physiol.* 234 19189–19198. 10.1002/jcp.28636 30980400

[B53] Hodkinson P. S. Elliott T. Wong W. S. Rintoul R. C. Mackinnon A. C. Haslett C. (2006). ECM overrides DNA damage-induced cell cycle arrest and apoptosis in small-cell lung cancer cells through beta1 integrin-dependent activation of PI3-kinase. *Cell Death Differ.* 13 1776–1788. 10.1038/sj.cdd.4401849 16410797

[B54] Hodkinson P. S. Mackinnon A. C. Sethi T. (2007). Extracellular matrix regulation of drug resistance in small-cell lung cancer. *Int. J. Radiat. Biol.* 83 733–741. 10.1080/09553000701570204 17852559

[B55] Hoellenriegel J. Meadows S. A. Sivina M. Wierda W. G. Kantarjian H. Keating M. J. (2011). The phosphoinositide 3’-kinase delta inhibitor, CAL-101, inhibits B-cell receptor signaling and chemokine networks in chronic lymphocytic leukemia. *Blood* 118 3603–3612. 10.1182/blood-2011-05-352492 21803855 PMC4916562

[B56] Hou S. Isaji T. Hang Q. Im S. Fukuda T. Gu J. (2016). Distinct effects of beta1 integrin on cell proliferation and cellular signaling in MDA-MB-231 breast cancer cells. *Sci. Rep.* 6:18430. 10.1038/srep18430 26728650 PMC4700444

[B57] Huang X. Wang Y. Nan X. He S. Xu X. Zhu X. (2014). The role of the orphan G protein-coupled receptor 37 (GPR37) in multiple myeloma cells. *Leuk. Res.* 38 225–235. 10.1016/j.leukres.2013.11.007 24290813

[B58] Huang Y. Huang X. Cheng C. Xu X. Liu H. Yang X. (2019). Elucidating the expression and function of Numbl during cell adhesion-mediated drug resistance (CAM-DR) in multiple myeloma (MM). *BMC Cancer* 19:1269. 10.1186/s12885-019-6446-y 31888545 PMC6937660

[B59] Huang Y. Xu X. Ji L. Wang Y. Wang S. Tang J. (2016). Expression of far upstream element binding protein 1 in Bcell nonHodgkin lymphoma is correlated with tumor growth and celladhesion mediated drug resistance. *Mol. Med. Rep.* 14 3759–3768. 10.3892/mmr.2016.5718 27599538

[B60] Hynes R. O. (1992). Integrins: versatility, modulation, and signaling in cell adhesion. *Cell* 69 11–25. 10.1016/0092-8674(92)90115-s 1555235

[B61] Ivanov D. B. Philippova M. P. Tkachuk V. A. (2001). Structure and functions of classical cadherins. *Biochemistry* 66 1174–1186. 10.1023/a:1012445316415 11736639

[B62] Jakubzig B. Baltes F. Henze S. Schlesinger M. Bendas G. (2018). Mechanisms of matrix-induced chemoresistance of breast cancer cells-deciphering novel potential targets for a cell sensitization. *Cancers* 10:495. 10.3390/cancers10120495 30563275 PMC6315379

[B63] Jeanes A. I. Wang P. Moreno-Layseca P. Paul N. Cheung J. Tsang R. (2012). Specific beta-containing integrins exert differential control on proliferation and two-dimensional collective cell migration in mammary epithelial cells. *J. Biol. Chem.* 287 24103–24112. 10.1074/jbc.M112.360834 22511753 PMC3397837

[B64] Jiang Y. Prosper F. Verfaillie C. M. (2000a). Opposing effects of engagement of integrins and stimulation of cytokine receptors on cell cycle progression of normal human hematopoietic progenitors. *Blood* 95 846–854. 10.1182/blood-2009-06-226373 10648395

[B65] Jiang Y. Zhao R. C. Verfaillie C. M. (2000b). Abnormal integrin-mediated regulation of chronic myelogenous leukemia CD34+ cell proliferation: BCR/ABL up-regulates the cyclin-dependent kinase inhibitor, p27Kip, which is relocated to the cell cytoplasm and incapable of regulating cdk2 activity. *Proc. Natl. Acad. Sci. U.S.A.* 97 10538–10543. 10.1073/pnas.190104497 10973491 PMC27060

[B66] Karagiannidis I. Salataj E. Said Abu Egal E. Beswick E. J. (2021). G-CSF in tumors: aggressiveness, tumor microenvironment and immune cell regulation. *Cytokine* 142:155479. 10.1016/j.cyto.2021.155479 33677228 PMC8044051

[B67] Karakasheva T. A. Lin E. W. Tang Q. Qiao E. Waldron T. J. Soni M. (2018). IL-6 mediates cross-talk between tumor cells and activated fibroblasts in the tumor microenvironment. *Cancer Res.* 78 4957–4970. 10.1158/0008-5472.CAN-17-2268 29976575 PMC6125177

[B68] Keats J. J. Fonseca R. Chesi M. Schop R. Baker A. Chng W. J. (2007). Promiscuous mutations activate the noncanonical NF-kappaB pathway in multiple myeloma. *Cancer Cell* 12 131–144. 10.1016/j.ccr.2007.07.003 17692805 PMC2083698

[B69] Keizer R. J. Funahashi Y. Semba T. Wanders J. Beijnen J. H. Schellens J. H. (2011). Evaluation of alpha2-integrin expression as a biomarker for tumor growth inhibition for the investigational integrin inhibitor E7820 in preclinical and clinical studies. *AAPS J.* 13 230–239. 10.1208/s12248-011-9260-2 21387147 PMC3085714

[B70] Khot V. M. Salunkhe A. B. Pricl S. Bauer J. Thorat N. D. Townley H. (2021). Nanomedicine-driven molecular targeting, drug delivery, and therapeutic approaches to cancer chemoresistance. *Drug Discov. Today* 26 724–739. 10.1016/j.drudis.2020.12.016 33359624

[B71] Kikuchi J. Koyama D. Wada T. Izumi T. Hofgaard P. O. Bogen B. (2015). Phosphorylation-mediated EZH2 inactivation promotes drug resistance in multiple myeloma. *J. Clin. Invest.* 125 4375–4390. 10.1172/JCI80325 26517694 PMC4665777

[B72] Kim H. N. Ruan Y. Ogana H. Kim Y. M. (2020). Cadherins, selectins, and integrins in CAM-DR in leukemia. *Front. Oncol.* 10:592733. 10.3389/fonc.2020.592733 33425742 PMC7793796

[B73] Kobune M. Chiba H. Kato J. Kato K. Nakamura K. Kawano Y. (2007). Wnt3/RhoA/ROCK signaling pathway is involved in adhesion-mediated drug resistance of multiple myeloma in an autocrine mechanism. *Mol. Cancer Ther.* 6 1774–1784. 10.1158/1535-7163.MCT-06-0684 17575106

[B74] Krause G. Hassenruck F. Hallek M. (2018). Copanlisib for treatment of B-cell malignancies: the development of a PI3K inhibitor with considerable differences to idelalisib. *Drug Des. Dev. Ther.* 12 2577–2590. 10.2147/DDDT.S142406 30174412 PMC6109662

[B75] Kurland J. F. Kodym R. Story M. D. Spurgers K. B. McDonnell T. J. Meyn R. E. (2001). NF-kappaB1 (p50) homodimers contribute to transcription of the bcl-2 oncogene. *J. Biol. Chem.* 276 45380–45386. 10.1074/jbc.M108294200 11567031

[B76] Kurtova A. V. Tamayo A. T. Ford R. J. Burger J. A. (2009). Mantle cell lymphoma cells express high levels of CXCR4, CXCR5, and VLA-4 (CD49d): importance for interactions with the stromal microenvironment and specific targeting. *Blood* 113 4604–4613. 10.1182/blood-2008-10-185827 19228923 PMC4969050

[B77] Landowski T. H. Olashaw N. E. Agrawal D. Dalton W. S. (2003). Cell adhesion-mediated drug resistance (CAM-DR) is associated with activation of NF-kappa B (RelB/p50) in myeloma cells. *Oncogene* 22 2417–2421. 10.1038/sj.onc.1206315 12717418

[B78] Lee J. W. Chung H. Y. Ehrlich L. A. Jelinek D. F. Callander N. S. Roodman G. D. (2004). IL-3 expression by myeloma cells increases both osteoclast formation and growth of myeloma cells. *Blood* 103 2308–2315. 10.1182/blood-2003-06-1992 14615378

[B79] Li A. J. Dhanraj J. P. Lopes G. Parker J. L. (2021). Clinical trial risk in leukemia: biomarkers and trial design. *Hematol. Oncol.* 39 105–113. 10.1002/hon.2818 33078436

[B80] Li W. Liu Z. Zhao C. Zhai L. (2015). Binding of MMP-9-degraded fibronectin to beta6 integrin promotes invasion via the FAK-Src-related Erk1/2 and PI3K/Akt/Smad-1/5/8 pathways in breast cancer. *Oncol. Rep.* 34 1345–1352. 10.3892/or.2015.4103 26134759

[B81] Li Y. Wang Y. Wang Z. Yi D. Ma S. (2015). Racial differences in three major NHL subtypes: descriptive epidemiology. *Cancer Epidemiol.* 39 8–13. 10.1016/j.canep.2014.12.001 25560974 PMC4323749

[B82] Li Z. W. Dalton W. S. (2006). Tumor microenvironment and drug resistance in hematologic malignancies. *Blood Rev.* 20 333–342. 10.1016/j.blre.2005.08.003 16920238

[B83] Liang Y. Meleady P. Cleary I. McDonnell S. Connolly L. Clynes M. (2001). Selection with melphalan or paclitaxel (Taxol) yields variants with different patterns of multidrug resistance, integrin expression and in vitro invasiveness. *Eur. J. Cancer* 37 1041–1052. 10.1016/s0959-8049(01)00086-7 11334731

[B84] Lin J. Lwin T. Zhao J. J. Tam W. Choi Y. S. Moscinski L. C. (2011). Follicular dendritic cell-induced microRNA-mediated upregulation of PRDM1 and downregulation of BCL-6 in non-Hodgkin’s B-cell lymphomas. *Leukemia* 25 145–152. 10.1038/leu.2010.230 20966935 PMC3083119

[B85] Lin K. K. Harrell M. I. Oza A. M. Oaknin A. Ray-Coquard I. Tinker A. V. (2019). BRCA reversion mutations in circulating tumor DNA predict primary and acquired resistance to the PARP inhibitor rucaparib in high-grade ovarian carcinoma. *Cancer Discov.* 9 210–219. 10.1158/2159-8290.CD-18-0715 30425037

[B86] Liu H. Ding L. Shen Y. Zhong F. Wang Q. Xu X. (2016). RBQ3 participates in multiple myeloma cell proliferation, adhesion and chemoresistance. *Int. J. Biol. Macromol.* 91 115–122. 10.1016/j.ijbiomac.2016.05.050 27189701

[B87] Liu H. Guo D. Sha Y. Zhang C. Jiang Y. Hong L. (2020). ANXA7 promotes the cell cycle, proliferation and cell adhesion-mediated drug resistance of multiple myeloma cells by up-regulating CDC5L. *Aging* 12 11100–11115. 10.18632/aging.103326 32526706 PMC7346058

[B88] Liu L. Zhang H. Sun L. Gao Y. Jin H. Liang S. (2010). ERK/MAPK activation involves hypoxia-induced MGr1-Ag/37LRP expression and contributes to apoptosis resistance in gastric cancer. *Int. J. Cancer* 127 820–829. 10.1002/ijc.25098 19998339

[B89] Lwin T. Crespo L. A. Wu A. Dessureault S. Shu H. B. Moscinski L. C. (2009). Lymphoma cell adhesion-induced expression of B cell-activating factor of the TNF family in bone marrow stromal cells protects non-Hodgkin’s B lymphoma cells from apoptosis. *Leukemia* 23 170–177. 10.1038/leu.2008.266 18843286

[B90] Lwin T. Hazlehurst L. A. Dessureault S. Lai R. Bai W. Sotomayor E. (2007a). Cell adhesion induces p27Kip1-associated cell-cycle arrest through down-regulation of the SCFSkp2 ubiquitin ligase pathway in mantle-cell and other non-Hodgkin B-cell lymphomas. *Blood* 110 1631–1638. 10.1182/blood-2006-11-060350 17502456 PMC1975846

[B91] Lwin T. Hazlehurst L. A. Li Z. Dessureault S. Sotomayor E. Moscinski L. C. (2007b). Bone marrow stromal cells prevent apoptosis of lymphoma cells by upregulation of anti-apoptotic proteins associated with activation of NF-kappaB (RelB/p52) in non-Hodgkin’s lymphoma cells. *Leukemia* 21 1521–1531. 10.1038/sj.leu.2404723 17476277

[B92] Lwin T. Lin J. Choi Y. S. Zhang X. Moscinski L. C. Wright K. L. (2010). Follicular dendritic cell-dependent drug resistance of non-Hodgkin lymphoma involves cell adhesion-mediated Bim down-regulation through induction of microRNA-181a. *Blood* 116 5228–5236. 10.1182/blood-2010-03-275925 20841506 PMC3012540

[B93] Malara A. Currao M. Gruppi C. Celesti G. Viarengo G. Buracchi C. (2014). Megakaryocytes contribute to the bone marrow-matrix environment by expressing fibronectin, type IV collagen, and laminin. *Stem Cells* 32 926–937. 10.1002/stem.1626 24357118 PMC4096110

[B94] Manier S. Sacco A. Leleu X. Ghobrial I. M. Roccaro A. M. (2012). Bone marrow microenvironment in multiple myeloma progression. *J. Biomed. Biotechnol.* 2012:157496. 10.1155/2012/157496 23093834 PMC3471001

[B95] Markman J. L. Rekechenetskiy A. Holler E. Ljubimova J. Y. (2013). Nanomedicine therapeutic approaches to overcome cancer drug resistance. *Adv. Drug Deliv. Rev.* 65 1866–1879. 10.1016/j.addr.2013.09.019 24120656 PMC5812459

[B96] Mensah F. A. Blaize J. P. Bryan L. J. (2018). Spotlight on copanlisib and its potential in the treatment of relapsed/refractory follicular lymphoma: evidence to date. *Oncol. Targets Ther.* 11 4817–4827. 10.2147/OTT.S142264 30147333 PMC6097514

[B97] Miao X. Wu Y. Wang Y. Zhu X. Yin H. He Y. (2016a). Y-box-binding protein-1 (YB-1) promotes cell proliferation, adhesion and drug resistance in diffuse large B-cell lymphoma. *Exp. Cell Res.* 346 157–166. 10.1016/j.yexcr.2016.07.003 27397581

[B98] Miao X. Xu X. Wu Y. Zhu X. Chen X. Li C. (2016b). Overexpression of TRIP6 promotes tumor proliferation and reverses cell adhesion-mediated drug resistance (CAM-DR) via regulating nuclear p27(Kip1) expression in non-Hodgkin’s lymphoma. *Tumour Biol.* 37 1369–1378. 10.1007/s13277-015-3939-4 26298725

[B99] Miller B. W. Przepiorka D. de Claro R. A. Lee K. Nie L. Simpson N. (2015). FDA approval: idelalisib monotherapy for the treatment of patients with follicular lymphoma and small lymphocytic lymphoma. *Clin. Cancer Res.* 21 1525–1529. 10.1158/1078-0432.CCR-14-2522 25645861

[B100] Miranti C. K. Brugge J. S. (2002). Sensing the environment: a historical perspective on integrin signal transduction. *Nat. Cell Biol.* 4 E83–E90. 10.1038/ncb0402-e83 11944041

[B101] Mita M. Kelly K. R. Mita A. Ricart A. D. Romero O. Tolcher A. (2011). Phase I study of E7820, an oral inhibitor of integrin alpha-2 expression with antiangiogenic properties, in patients with advanced malignancies. *Clin. Cancer Res.* 17 193–200. 10.1158/1078-0432.CCR-10-0010 21208908

[B102] Morishita N. Tsukahara H. Chayama K. Ishida T. Washio K. Miyamura T. (2012). Activation of Akt is associated with poor prognosis and chemotherapeutic resistance in pediatric B-precursor acute lymphoblastic leukemia. *Pediatr. Blood Cancer* 59 83–89. 10.1002/pbc.24034 22183914

[B103] Mraz M. Zent C. S. Church A. K. Jelinek D. F. Wu X. Pospisilova S. (2011). Bone marrow stromal cells protect lymphoma B-cells from rituximab-induced apoptosis and targeting integrin alpha-4-beta-1 (VLA-4) with natalizumab can overcome this resistance. *Br. J. Haematol.* 155 53–64. 10.1111/j.1365-2141.2011.08794.x 21749361 PMC4405035

[B104] Mudry R. E. Fortney J. E. York T. Hall B. M. Gibson L. F. (2000). Stromal cells regulate survival of B-lineage leukemic cells during chemotherapy. *Blood* 96 1926–1932. 10.1182/blood.V96.5.1926 10961896

[B105] Murray R. Z. Norbury C. (2000). Proteasome inhibitors as anti-cancer agents. *Anticancer Drugs* 11 407–417. 10.1097/00001813-200007000-00001 11001381

[B106] Naci D. Berrazouane S. Barabe F. Aoudjit F. (2019). Cell adhesion to collagen promotes leukemia resistance to doxorubicin by reducing DNA damage through the inhibition of Rac1 activation. *Sci. Rep.* 9:19455. 10.1038/s41598-019-55934-w 31857649 PMC6923425

[B107] Naci D. Vuori K. Aoudjit F. (2015). Alpha2beta1 integrin in cancer development and chemoresistance. *Semin. Cancer Biol.* 35 145–153. 10.1016/j.semcancer.2015.08.004 26297892

[B108] Nakagawa Y. Nakayama H. Nagata M. Yoshida R. Kawahara K. Hirosue A. (2014). Overexpression of fibronectin confers cell adhesion-mediated drug resistance (CAM-DR) against 5-FU in oral squamous cell carcinoma cells. *Int. J. Oncol.* 44 1376–1384. 10.3892/ijo.2014.2265 24452447

[B109] Nefedova Y. Landowski T. H. Dalton W. S. (2003). Bone marrow stromal-derived soluble factors and direct cell contact contribute to de novo drug resistance of myeloma cells by distinct mechanisms. *Leukemia* 17 1175–1182. 10.1038/sj.leu.2402924 12764386

[B110] Neri P. Ren L. Azab A. K. Brentnall M. Gratton K. Klimowicz A. C. (2011). Integrin beta7-mediated regulation of multiple myeloma cell adhesion, migration, and invasion. *Blood* 117 6202–6213. 10.1182/blood-2010-06-292243 21474670 PMC3122944

[B111] Nguyen L. X. Sesay A. Mitchell B. S. (2014). Effect of CAL-101, a PI3Kdelta inhibitor, on ribosomal rna synthesis and cell proliferation in acute myeloid leukemia cells. *Blood Cancer J.* 4:e228. 10.1038/bcj.2014.49 25014775 PMC4219447

[B112] No author list (2018). Duvelisib approved for leukemia, lymphoma. *Cancer Discov.* 8:OF4. 10.1158/2159-8290.CD-NB2018-137 30352859

[B113] Oshiro M. M. Landowski T. H. Catlett-Falcone R. Hazlehurst L. A. Huang M. Jove R. (2001). Inhibition of JAK kinase activity enhances Fas-mediated apoptosis but reduces cytotoxic activity of topoisomerase II inhibitors in U266 myeloma cells. *Clin. Cancer Res.* 7 4262–4271. 11751528

[B114] Ouyang Y. Zhong F. Wang Q. Ding L. Zhang P. Chen L. (2016). DIXDC1 promotes tumor proliferation and cell adhesion mediated drug resistance (CAM-DR) via enhancing p-Akt in Non-Hodgkin’s lymphomas. *Leuk. Res.* 50 104–111. 10.1016/j.leukres.2016.09.011 27701018

[B115] Prakash J. de Jong E. Post E. Gouw A. S. Beljaars L. Poelstra K. (2010). A novel approach to deliver anticancer drugs to key cell types in tumors using a PDGF receptor-binding cyclic peptide containing carrier. *J. Control Release* 145 91–101. 10.1016/j.jconrel.2010.03.018 20362019

[B116] Ragon B. K. Kantarjian H. Jabbour E. Ravandi F. Cortes J. Borthakur G. (2017). Buparlisib, a PI3K inhibitor, demonstrates acceptable tolerability and preliminary activity in a phase I trial of patients with advanced leukemias. *Am. J. Hematol.* 92 7–11. 10.1002/ajh.24568 27673440 PMC5361214

[B117] Rainero E. Norman J. C. (2015). Endosomal integrin signals for survival. *Nat. Cell Biol.* 17 1373–1375. 10.1038/ncb3261 26515017

[B118] Rintoul R. C. Sethi T. (2001). The role of extracellular matrix in small-cell lung cancer. *Lancet Oncol.* 2 437–442. 10.1016/S1470-2045(00)00421-6 11905738

[B119] Roboz G. J. Jabbour E. J. Faderl S. Douer D. (2014). Advances in the treatment of relapsed/refractory acute lymphoblastic leukemia: a case study compendium. *Clin. Adv. Hematol. Oncol.* 12 8–18. 25768269

[B120] Ruan Y. Kim H. N. Ogana H. Kim Y. M. (2020). Wnt signaling in leukemia and its bone marrow microenvironment. *Int. J. Mol. Sci.* 21:6247. 10.3390/ijms21176247 32872365 PMC7503842

[B121] Said G. Guilbert M. Morjani H. Garnotel R. Jeannesson P. El Btaouri H. (2012). Extracellular matrix proteins modulate antimigratory and apoptotic effects of Doxorubicin. *Chemother. Res. Pract.* 2012:268681. 10.1155/2012/268681 22811904 PMC3395309

[B122] Saito R. Amemiya H. Hosomura N. Kawaida H. Maruyama S. Shimizu H. (2019). Prognostic factors for post-recurrent survival in hepatocellular carcinoma after curative resection. *Anticancer Res.* 39 3033–3038. 10.21873/anticanres.13436 31177145

[B123] Sanchez V. E. Nichols C. Kim H. N. Gang E. J. Kim Y. M. (2019). Targeting PI3K signaling in acute lymphoblastic leukemia. *Int. J. Mol. Sci.* 20:412. 10.3390/ijms20020412 30669372 PMC6358886

[B124] Sethi T. Rintoul R. C. Moore S. M. MacKinnon A. C. Salter D. Choo C. (1999). Extracellular matrix proteins protect small cell lung cancer cells against apoptosis: a mechanism for small cell lung cancer growth and drug resistance in vivo. *Nat. Med.* 5 662–668. 10.1038/9511 10371505

[B125] Sevilla-Movilla S. Arellano-Sanchez N. Martinez-Moreno M. Gajate C. Sanchez-Vencells A. Valcarcel L. V. (2020). Upregulated expression and function of the alpha4beta1 integrin in multiple myeloma cells resistant to bortezomib. *J. Pathol.* 252 29–40. 10.1002/path.5480 32501543

[B126] Shain K. H. Landowski T. H. Dalton W. S. (2002). Adhesion-mediated intracellular redistribution of c-Fas-associated death domain-like IL-1-converting enzyme-like inhibitory protein-long confers resistance to CD95-induced apoptosis in hematopoietic cancer cell lines. *J. Immunol.* 168 2544–2553. 10.4049/jimmunol.168.5.2544 11859150

[B127] Shao S. Huang X. Wang Y. He S. Xu X. Zhu X. (2014). A role for activator of G-protein signaling 3 (AGS3) in multiple myeloma. *Int. J. Hematol.* 99 57–68. 10.1007/s12185-013-1484-8 24307516

[B128] Shishido S. Bonig H. Kim Y. M. (2014). Role of integrin alpha4 in drug resistance of leukemia. *Front. Oncol.* 4:99. 10.3389/fonc.2014.00099 24904821 PMC4033044

[B129] Singh R. R. Kunkalla K. Qu C. Schlette E. Neelapu S. S. Samaniego F. (2011). ABCG2 is a direct transcriptional target of hedgehog signaling and involved in stroma-induced drug tolerance in diffuse large B-cell lymphoma. *Oncogene* 30 4874–4886. 10.1038/onc.2011.195 21625222 PMC3165099

[B130] Song Y. Kim J. S. Choi E. K. Kim J. Kim K. M. Seo H. R. (2017). TGF-beta-independent CTGF induction regulates cell adhesion mediated drug resistance by increasing collagen I in HCC. *Oncotarget* 8 21650–21662. 10.18632/oncotarget.15521 28423507 PMC5400613

[B131] Stefanzl G. Berger D. Cerny-Reiterer S. Blatt K. Eisenwort G. Sperr W. R. (2017). The pan-BCL-2-blocker obatoclax (GX15-070) and the PI3-kinase/mTOR-inhibitor BEZ235 produce cooperative growth-inhibitory effects in ALL cells. *Oncotarget* 8 67709–67722. 10.18632/oncotarget.18810 28978065 PMC5620205

[B132] Sun L. Liu L. Liu X. Wang Y. Li M. Yao L. (2014a). Gastric cancer cell adhesion to laminin enhances acquired chemotherapeutic drug resistance mediated by MGr1-Ag/37LRP. *Oncol. Rep.* 32 105–114. 10.3892/or.2014.3184 24840404

[B133] Sun L. Liu L. Liu X. Wang Y. Li M. Yao L. (2014b). MGr1-Ag/37LRP induces cell adhesion-mediated drug resistance through FAK/PI3K and MAPK pathway in gastric cancer. *Cancer Sci.* 105 651–659. 10.1111/cas.12414 24703465 PMC4317895

[B134] Takeda T. Tsubak M. Genno S. Matsuda T. Yamamoto Y. Ueda E. (2020). CD49d and CD49e induce cell adhesion-mediated drug resistance through the nuclear factor-kappaB pathway in Burkitt lymphoma. *J. Physiol. Pharmacol.* 71. 10.26402/jpp.2020.4.02 [Epub ahead of print]. 33214335

[B135] Tang J. Ji L. Wang Y. Huang Y. Yin H. He Y. (2015). Cell adhesion down-regulates the expression of vacuolar protein sorting 4B (VPS4B) and contributes to drug resistance in multiple myeloma cells. *Int. J. Hematol.* 102 25–34. 10.1007/s12185-015-1783-3 25804841

[B136] Tang J. Zhou H. Wang C. Fei X. Zhu L. Huang Y. (2016). Cell adhesion downregulates the expression of Homer1b/c and contributes to drug resistance in multiple myeloma cells. *Oncol. Rep.* 35 1875–1883. 10.3892/or.2015.4532 26718835

[B137] Tang L. A. Dixon B. N. Maples K. T. Poppiti K. M. Peterson T. J. (2018). Current and investigational agents targeting the phosphoinositide 3-kinase pathway. *Pharmacotherapy* 38 1058–1067. 10.1002/phar.2173 30120858

[B138] Thompson C. A. Purushothaman A. Ramani V. C. Vlodavsky I. Sanderson R. D. (2013). Heparanase regulates secretion, composition, and function of tumor cell-derived exosomes. *J. Biol. Chem.* 288 10093–10099. 10.1074/jbc.C112.444562 23430739 PMC3617250

[B139] Tjin E. P. Groen R. W. Vogelzang I. Derksen P. W. Klok M. D. Meijer H. P. (2006). Functional analysis of HGF/MET signaling and aberrant HGF-activator expression in diffuse large B-cell lymphoma. *Blood* 107 760–768. 10.1182/blood-2005-05-1929 16189274

[B140] Toth R. K. Tran J. D. Muldong M. T. Nollet E. A. Schulz V. V. Jensen C. C. (2019). Hypoxia-induced PIM kinase and laminin-activated integrin alpha6 mediate resistance to PI3K inhibitors in bone-metastatic CRPC. *Am. J. Clin. Exp. Urol.* 7 297–312. 31511835 PMC6734039

[B141] Ullah T. R. (2019). The role of CXCR4 in multiple myeloma: cells’ journey from bone marrow to beyond. *J. Bone Oncol.* 17 100253. 10.1016/j.jbo.2019.100253 31372333 PMC6658931

[B142] Vedenko A. Panara K. Goldstein G. Ramasamy R. Arora H. (2020). Tumor microenvironment and nitric oxide: concepts and mechanisms. *Adv. Exp. Med. Biol.* 1277 143–158. 10.1007/978-3-030-50224-9_10 33119871

[B143] Vella V. Malaguarnera R. Nicolosi M. L. Morrione A. Belfiore A. (2019). Insulin/IGF signaling and discoidin domain receptors: an emerging functional connection. *Biochim. Biophys. Acta Mol. Cell Res.* 1866:118522. 10.1016/j.bbamcr.2019.118522 31394114

[B144] Waldschmidt J. M. Simon A. Wider D. Muller S. J. Follo M. Ihorst G. (2017). CXCL12 and CXCR7 are relevant targets to reverse cell adhesion-mediated drug resistance in multiple myeloma. *Br. J. Haematol.* 179 36–49. 10.1111/bjh.14807 28670693

[B145] Wang J. Liu X. Qiu Y. Shi Y. Cai J. Wang B. (2018). Cell adhesion-mediated mitochondria transfer contributes to mesenchymal stem cell-induced chemoresistance on T cell acute lymphoblastic leukemia cells. *J. Hematol. Oncol.* 11:11. 10.1186/s13045-018-0554-z 29357914 PMC5778754

[B146] Wang K. Jiang Y. Zheng W. Liu Z. Li H. Lou J. (2013). Silencing of human phosphatidylethanolamine-binding protein 4 enhances rituximab-induced death and chemosensitization in B-cell lymphoma. *PLoS One* 8:e56829. 10.1371/journal.pone.0056829 23451095 PMC3581549

[B147] Wang M. R. Chen R. J. Zhao F. Zhang H. H. Bi Q. Y. Zhang Y. N. (2020). Effect of wenxia changfu formula combined with cisplatin reversing non-small cell lung cancer cell adhesion-mediated drug resistance. *Front. Pharmacol.* 11:500137. 10.3389/fphar.2020.500137 33041787 PMC7527591

[B148] Wang W. Wei R. Liu S. Qiao L. Hou J. Gu C. (2019). BTK induces CAM-DR through regulation of CXCR4 degradation in multiple myeloma. *Am. J. Transl. Res.* 11 4139–4150. 31396324 PMC6684885

[B149] Wang X. Li C. Ju S. Wang Y. Wang H. Zhong R. (2011). Myeloma cell adhesion to bone marrow stromal cells confers drug resistance by microRNA-21 up-regulation. *Leuk. Lymphoma* 52 1991–1998. 10.3109/10428194.2011.591004 21718132

[B150] Wang Y. Huang Y. Xu X. Tang J. Huang X. Zhu J. (2014). Expression of small glutamine-rich TPR-containing protein A (SGTA) in Non-Hodgkin’s Lymphomas promotes tumor proliferation and reverses cell adhesion-mediated drug resistance (CAM-DR). *Leuk. Res.* 38 955–963. 10.1016/j.leukres.2014.05.013 24974147

[B151] Wang Y. Wu Y. Miao X. Zhu X. He Y. Zhong F. (2015). Silencing of DYRK2 increases cell proliferation but reverses CAM-DR in Non-Hodgkin’s Lymphoma. *Int. J. Biol. Macromol.* 81 809–817. 10.1016/j.ijbiomac.2015.08.067 26341817

[B152] Westhoff M. A. Zhou S. Bachem M. G. Debatin K. M. Fulda S. (2008). Identification of a novel switch in the dominant forms of cell adhesion-mediated drug resistance in glioblastoma cells. *Oncogene* 27 5169–5181. 10.1038/onc.2008.148 18469856

[B153] Willenbacher W. Seeber A. Steiner N. Willenbacher E. Gatalica Z. Swensen J. (2018). Towards molecular profiling in multiple myeloma: a literature review and early indications of its efficacy for informing treatment strategies. *Int. J. Mol. Sci.* 19:2087. 10.3390/ijms19072087 30021955 PMC6073692

[B154] Wu Y. Xu X. Miao X. Zhu X. Yin H. He Y. (2015). Sam68 regulates cell proliferation and cell adhesion-mediated drug resistance (CAM-DR) via the AKT pathway in non-Hodgkin’s lymphoma. *Cell Prolif.* 48 682–690. 10.1111/cpr.12220 26478515 PMC6495998

[B155] Wu Y. Zhang J. Li Q. (2021). Autophagy, an accomplice or antagonist of drug resistance in HCC? *Cell Death Dis.* 12:266. 10.1038/s41419-021-03553-7 33712559 PMC7954824

[B156] Wu Y. Zhu X. Shen R. Huang J. Xu X. He S. (2019). miR-182 contributes to cell adhesion-mediated drug resistance in multiple myeloma via targeting PDCD4. *Pathol. Res. Pract.* 215:152603. 10.1016/j.prp.2019.152603 31540771

[B157] Xargay-Torrent S. Lopez-Guerra M. Montraveta A. Saborit-Villarroya I. Rosich L. Navarro A. (2013). Sorafenib inhibits cell migration and stroma-mediated bortezomib resistance by interfering B-cell receptor signaling and protein translation in mantle cell lymphoma. *Clin. Cancer Res.* 19 586–597. 10.1158/1078-0432.CCR-12-1935 23231952

[B158] Xu X. He Y. Miao X. Wu Y. Han J. Wang Q. (2016). Cell adhesion induces overexpression of chromodomain helicase/ATPase DNA binding protein 1-like gene (CHD1L) and contributes to cell adhesion-mediated drug resistance (CAM-DR) in multiple myeloma cells. *Leuk. Res.* 47 54–62. 10.1016/j.leukres.2016.05.007 27258734

[B159] Xu X. Wang Q. He Y. Ding L. Zhong F. Ou Y. (2017). ADP-ribosylation factor 1 (ARF1) takes part in cell proliferation and cell adhesion-mediated drug resistance (CAM-DR). *Ann. Hematol.* 96 847–858. 10.1007/s00277-017-2949-2 28238095

[B160] Yagi K. Yamamoto K. Umeda S. Abe S. Suzuki S. Onishi I. (2013). Expression of multidrug resistance 1 gene in B-cell lymphomas: association with follicular dendritic cells. *Histopathology* 62 414–420. 10.1111/his.12035 23339364

[B161] Yan L. Wang C. Lin B. Liu J. Liu D. Hou R. (2015). Lewis y enhances CAM-DR in ovarian cancer cells by activating the FAK signaling pathway and upregulating Bcl-2/Bcl-XL expression. *Biochimie* 113 17–25. 10.1016/j.biochi.2015.01.013 25726913

[B162] Yang R. Huo Z. Duan Y. Tong W. Zheng Y. Su Y. (2020). SOX11 inhibits tumor proliferation and promotes cell adhesion mediated-drug resistance via a CD43 dependent manner in mantle cell lymphoma. *Leuk. Lymphoma* 61 2068–2081. 10.1080/10428194.2020.1762877 32449421

[B163] Yin H. Zhong F. Ouyang Y. Wang Q. Ding L. He S. (2017). Upregulation of ADAM12 contributes to accelerated cell proliferation and cell adhesion-mediated drug resistance (CAM-DR) in Non-Hodgkin’s Lymphoma. *Hematology* 22 527–535. 10.1080/10245332.2017.1312205 28395594

[B164] Zhan F. Colla S. Wu X. Chen B. Stewart J. P. Kuehl W. M. (2007). CKS1B, overexpressed in aggressive disease, regulates multiple myeloma growth and survival through SKP2- and p27Kip1-dependent and -independent mechanisms. *Blood* 109 4995–5001. 17303695 10.1182/blood-2006-07-038703 PMC1885527

[B165] Zhang B. Li M. McDonald T. Holyoake T. L. Moon R. T. Campana D. (2013). Microenvironmental protection of CML stem and progenitor cells from tyrosine kinase inhibitors through N-cadherin and Wnt-beta-catenin signaling. *Blood* 121 1824–1838. 10.1182/blood-2012-02-412890 23299311 PMC3591802

[B166] Zhang P. P. Wang Y. C. Cheng C. Zhang F. Ding D. Z. Chen D. K. (2020). Runt-related transcription factor 2 influences cell adhesion-mediated drug resistance and cell proliferation in B-cell non-Hodgkin’s lymphoma and multiple myeloma. *Leuk. Res.* 92:106340. 10.1016/j.leukres.2020.106340 32182487

[B167] Zhang P. Zhang C. Li J. Han J. Liu X. Yang H. (2019). The physical microenvironment of hematopoietic stem cells and its emerging roles in engineering applications. *Stem Cell Res. Ther.* 10:327. 10.1186/s13287-019-1422-7 31744536 PMC6862744

[B168] Zhao M. Tao F. Venkatraman A. Li Z. Smith S. E. Unruh J. (2019). N-Cadherin-expressing bone and marrow stromal progenitor cells maintain reserve hematopoietic stem cells. *Cell Rep.* 26 652–669.e6. 10.1016/j.celrep.2018.12.093 30650358 PMC6890378

[B169] Zhu B. Zhao L. Zhu L. Wang H. Sha Y. Yao J. (2012). Oroxylin A reverses CAM-DR of HepG2 cells by suppressing Integrinbeta1 and its related pathway. *Toxicol. Appl. Pharmacol.* 259 387–394. 10.1016/j.taap.2012.01.019 22310179

[B170] Zhu L. C. Gao J. Hu Z. H. Schwab C. L. Zhuang H. Y. Tan M. Z. (2015). Membranous expressions of Lewis y and CAM-DR-related markers are independent factors of chemotherapy resistance and poor prognosis in epithelial ovarian cancer. *Am. J. Cancer Res.* 5 830–843. 25973320 PMC4396026

[B171] Zhu X. Miao X. Wu Y. Li C. Guo Y. Liu Y. (2015). ENO1 promotes tumor proliferation and cell adhesion mediated drug resistance (CAM-DR) in Non-Hodgkin’s Lymphomas. *Exp. Cell Res.* 335 216–223. 10.1016/j.yexcr.2015.05.020 26024773

[B172] Zhu X. Ouyang Y. Zhong F. Wang Q. Ding L. Zhang P. (2017). Silencing of CKIP-1 promotes tumor proliferation and cell adhesion-mediated drug resistance via regulating AKT activity in non-Hodgkin’s lymphoma. *Oncol. Rep.* 37 622–630. 10.3892/or.2016.5233 27840970

